# Anti-QS Strategies Against *Pseudomonas aeruginosa* Infections

**DOI:** 10.3390/microorganisms13081838

**Published:** 2025-08-07

**Authors:** Abdelaziz Touati, Nasir Adam Ibrahim, Lilia Tighilt, Takfarinas Idres

**Affiliations:** 1 Laboratory of Microbial Ecology, Faculté des Sciences de la Nature et de la Vie (FSNV), University of Bejaia, Bejaia 06000, Algeria; lilia.tighilt@univ-bejaia.dz; 2 Department of Biology, College of Science, Imam Mohammad Ibn Saud Islamic University (IMSIU), Riyadh 13318, Saudi Arabia; naabdalneim@imamu.edu.sa; 3 Laboratory for Livestock Animal Production and Health Research, Rabie Bouchama National Veterinary School of Algiers, Issad ABBAS Street, BP 161 Oued Smar, Algiers 16059, Algeria; t.idres@ensv.dz

**Keywords:** *Pseudomonas aeruginosa*, quorum-sensing inhibition, antibiotic resistance, biofilm disruption, antivirulence therapy, bacterial communication

## Abstract

*Pseudomonas aeruginosa* poses significant health threats due to its multidrug-resistant profile, particularly affecting immunocompromised individuals. The pathogen’s ability to produce virulence factors and antibiotic-resistant biofilms, orchestrated through quorum-sensing (QS) mechanisms, complicates conventional therapeutic interventions. This review aims to critically assess the potential of anti-QS strategies as alternatives to antibiotics against *P. aeruginosa* infections. Comprehensive literature searches were conducted using databases such as PubMed, Scopus, and Web of Science, focusing on studies addressing QS inhibition strategies published recently. Anti-QS strategies significantly attenuate bacterial virulence by disrupting QS-regulated genes involved in biofilm formation, motility, toxin secretion, and immune evasion. These interventions reduce the selective pressure for resistance and enhance antibiotic efficacy when used in combination therapies. Despite promising outcomes, practical application faces challenges, including specificity of inhibitors, pharmacokinetic limitations, potential cytotoxicity, and bacterial adaptability leading to resistance. Future perspectives should focus on multi-target QS inhibitors, advanced delivery systems, rigorous preclinical validations, and clinical translation frameworks. Addressing current limitations through multidisciplinary research can lead to clinically viable QS-targeted therapies, offering sustainable alternatives to traditional antibiotics and effectively managing antibiotic resistance.

## 1. Introduction

*Pseudomonas aeruginosa* is a formidable opportunistic pathogen responsible for severe infections, particularly in immunocompromised individuals, such as those with cystic fibrosis (CF), burns, or immunosuppressive conditions [1]. Its clinical significance stems from its ability to produce diverse virulence factors and form antibiotic-resistant biofilms, rendering conventional therapies ineffective. The rise of multidrug-resistant (MDR) strains due to antibiotic overuse underscores the urgent need for alternative therapeutic strategies [2].

The pathogenicity of *P. aeruginosa* is mediated by an arsenal of virulence factors, including pyocyanin, pyoverdin, elastase, rhamnolipids, and proteases, which collectively damage host tissues, evade immune responses, and facilitate nutrient acquisition. These factors are tightly regulated by quorum sensing (QS), a cell-density-dependent signaling system that coordinates bacterial behavior [3]. The *las* and *rhl* QS systems, governed by the *lasI/lasR* and *rhlI/rhlR* genes, regulate biofilm formation, motility, and toxin secretion. QS ensures synchronized expression of virulence determinants, enabling the bacteria to adapt dynamically to host environments [4]. For instance, biofilm formation, a hallmark of chronic infections, is intrinsically linked to QS activation, allowing bacterial communities to adhere to surfaces and resist clearance [5].

*P. aeruginosa* exhibits intrinsic and acquired resistance to multiple antibiotics, including β-lactams, fluoroquinolones, and aminoglycosides, due to efflux pumps, enzymatic inactivation, and membrane permeability alterations [2]. However, biofilm formation represents a critical resistance mechanism, as extracellular polymeric substances (EPS) in biofilms physically shield bacteria from antibiotics and host immune cells. Biofilms also create metabolic gradients, inducing dormant persister cells that tolerate high antibiotic concentrations [6,7]. Genetic and biofilm—mediated dual resistance strategy renders *P. aeruginosa* infections recurrent and challenging to eradicate, particularly in hospital-acquired pneumonia, urinary tract infections, and chronic wounds [8].

To circumvent antibiotic resistance, novel strategies targeting virulence and biofilm formation without exerting bactericidal pressure are under investigation [9]. Anti-QS therapies disrupt bacterial communication, attenuating virulence factor production and biofilm development. Natural and synthetic QS inhibitors (QSIs), such as plant-derived compounds and repurposed drugs, have shown promise in downregulating *las* and *rhl* systems [10,11]. For example, plant extracts rich in polyphenols and alkaloids inhibit QS-regulated genes, reducing pyocyanin synthesis and biofilm biomass [12]. Similarly, silver nanoparticles synthesized via green chemistry exhibit dual antibacterial and anti-QS activity, destabilizing biofilms through oxidative stress and interference with QS signal molecules [13].

Complementary approaches include bacteriophage therapy, which selectively lyses biofilms while preserving commensal microbiota, and hybrid nanoparticle systems that enhance antibiotic delivery [14]. Hybrid solid lipid nanoparticles (SLNs) co-loaded with antibiotics and natural antimicrobials improve drug solubility and biofilm penetration, synergistically reducing bacterial viability [15]. Marine-derived bioactive compounds, such as actinobacterial metabolites and mollusk-derived peptides, disrupt biofilm architecture and QS signaling pathways [16]. High-throughput screening of FDA-approved drugs has further identified candidates that suppress QS-regulated virulence genes, offering rapid translational potential [17].

The main objective of this review article is to provide a structured overview that begins with a concise examination of the virulence factors of *P. aeruginosa* and their clinical implications. Subsequently, this review discusses various QSIs, categorized according to their sources, including natural products, synthetic compounds, and bioengineered molecules. Furthermore, the review identifies existing limitations and challenges related to the practical therapeutic application of QSIs. It concludes by outlining future perspectives and suggesting potential research avenues that may facilitate advancements in the development and clinical implementation of QS inhibition strategies. It is important to note that although the selection of QSIs presented herein is extensive, it is not exhaustive; nonetheless, considerable effort has been made to encompass a broad representation of the relevant literature.

## 2. Virulence Mechanisms of *P. aeruginosa*

*P. aeruginosa* is a highly adaptable opportunistic pathogen that utilizes a variety of virulence mechanisms to establish and persist in host environments, particularly in immunocompromised individuals and patients with chronic respiratory diseases such as CF. This bacterium employs diverse strategies, including biofilm formation, QS, motility mechanisms, secretion systems, and the production of a wide range of toxins, collectively contributing to its pathogenicity and resistance to host immune defenses and antimicrobial agents.

### 2.1. Biofilm Formation and Antibiotic Resistance in P. aeruginosa

A defining virulence feature of *P. aeruginosa* is its capacity to form biofilms—structured microbial communities encased in a self-produced extracellular matrix. This matrix consists of polysaccharides (e.g., Psl, Pel, and alginate), extracellular DNA (eDNA), proteins, and lipids, which collectively shield bacteria from environmental stressors, host immune defenses, and antimicrobial agents [5,18]. The biofilm’s physical integrity is further reinforced by adhesins such as type IV pili and flagella, which mediate surface attachment, and components like rhamnolipids and amyloid-like fimbriae (ALF) encoded by the *fap* operon, which enhance stability and resistance to environmental perturbations [19,20,21].

Biofilm-mediated chronic infections are particularly detrimental in CF, where *P. aeruginosa* adopts a mucoid phenotype characterized by alginate overproduction [22,23]. This phenotype exacerbates respiratory failure by promoting bacterial persistence, impairing mucociliary clearance, and evading antibiotic penetration and host immune responses [24]. Beyond CF, biofilms contribute to the recalcitrance of wound infections, urinary tract infections, and device-associated infections (e.g., ventilators, catheters), where surface-adherent communities resist clearance and prolong host tissue damage [5,25].

Biofilm formation is tightly regulated by QS, a cell-density-dependent signaling system that coordinates the expression of virulence factors, including matrix components and adhesins [18,26]. This regulatory network enhances biofilm maturation and adaptability, further complicating treatment. The combined effects of the EPS matrix, phenotypic diversification, and QS-driven gene expression render biofilm-associated infections extraordinarily resistant to conventional antibiotics [27]. For instance, eDNA within the matrix stabilizes biofilm architecture and binds antimicrobial agents, reducing their efficacy [24]. Consequently, biofilm-related infections often necessitate prolonged, high-dose antibiotic regimens or device removal, underscoring the urgent need for novel therapeutic strategies targeting biofilm dispersal or matrix disruption [28].

### 2.2. QS and Virulence Regulation in P. aeruginosa

**Figure 1:**
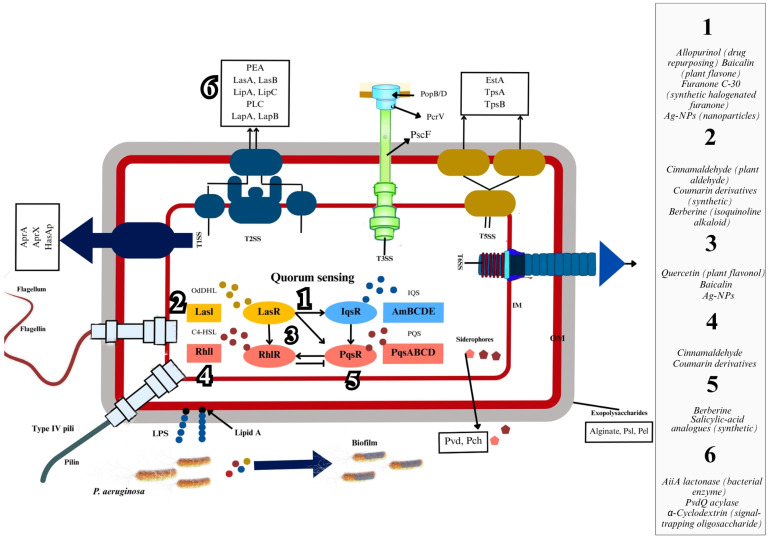
QS and virulence mechanisms in *P. aeruginosa.* Legend. This image highlights the various QS systems (LasI/LasR, RhlI/RhlR, IqsR) that regulate the production of virulence factors such as exopolysaccharides (alginate, Psl, Pel), type IV pili, and siderophores. The image also shows the secretion systems (T2SS, T3SS) involved in the export of toxins and other virulence factors, such as PopB/D and PcrV. The interaction between QS signals, such as OdDHL and C4-HSL, leads to the activation of these systems, facilitating the pathogen’s ability to form biofilms and cause infections. This mechanism is central to the pathogenicity of *P. aeruginosa*, contributing to its persistence and resistance in host environments.

*P. aeruginosa* employs QS to coordinate virulence factor production and population-wide behaviors. Four interconnected QS systems—Las, Rhl, *Pseudomonas* quinolone signal (PQS), and integrated QS (Iqs)—govern this regulatory network [24,29,30]. These systems rely on autoinducers, small signaling molecules that synchronize gene expression across bacterial populations, enabling adaptive responses to environmental changes [31]. QS controls the expression of diverse virulence determinants critical for infection (Figure 1): Toxins and Proteases: Exotoxins (ExoS, ExoT, ExoU, ExoY), elastases (LasA, LasB), and proteases contribute to tissue damage, host cell disruption, and immune evasion [31,32].

Biofilm Components: QS regulates exopolysaccharides and rhamnolipids, which stabilize biofilm architecture, enhance bacterial persistence, and confer resistance to host defenses [5,33].

Iron Acquisition and Secondary Metabolites: Pyocyanin and pyoverdine, regulated by QS, facilitate iron scavenging and oxidative stress induction, further compromising host immunity [34].

Secretion Systems: The Type VI secretion system (T6SS), linked to the *Pseudomonas* virulence factor (pvf) gene cluster, mediates host invasion and interbacterial competition [32,35].

The QS network integrates approximately 70 transcription factors (TFs), including Las, Rhl, Pqs, and Iqs, to dynamically adjust virulence gene expression [33]. This hierarchical regulation ensures precise control over biofilm maturation, motility, and toxin secretion. For instance, the *pvf* cluster synthesizes autoinducers that activate T6SS components and protease production, amplifying pathogenicity [32].

QS-driven adaptations significantly enhance the ability of *P. aeruginosa* to thrive across diverse ecological niches. Through immune evasion and tissue damage, QS-controlled factors like rhamnolipids and elastases actively degrade host cell membranes, while exotoxins disrupt vital cellular processes [29,31]. Furthermore, antibiotic resistance in *P. aeruginosa* is bolstered by QS-regulated biofilm formation and efflux pump activity, collectively reducing antimicrobial susceptibility and complicating treatment efforts [24,28]. Lastly, QS signaling pathways facilitate environmental adaptability, allowing rapid transitions between acute virulent states characterized by toxin release and chronic persistence phases through biofilm formation, ensuring survival in fluctuating conditions [19,36].

### 2.3. Motility Mechanisms and Host Colonization in P. aeruginosa

*P. aeruginosa* employs diverse motility mechanisms, including flagellar-mediated swimming and type IV pili (T4P)-dependent twitching motility, to colonize host tissues and establish infections [37]. These systems facilitate surface attachment, tissue penetration, and biofilm formation, which are critical for acute and chronic infections [18,38]. Flagella, composed of structural proteins such as FliC (flagellin) and FliD (filament cap), enable swimming motility through liquid environments, allowing the bacterium to navigate toward favorable niches [39]. Conversely, T4P—governed by components including PilA (pilin monomer), PilB (assembly ATPase), PilT, and PilU (retraction ATPases)—mediate twitching motility, a surface-associated movement essential for microcolony formation, biofilm initiation, and host tissue colonization [30,40].

The transition between planktonic and biofilm-associated lifestyles is dynamically regulated by intracellular signaling molecules such as cyclic di-GMP (c-di-GMP) and cAMP. Elevated c-di-GMP levels promote biofilm formation by suppressing motility, while reduced concentrations favor dispersal and motility [41]. Beyond physical movement, these motility structures act as pathogen-associated molecular patterns (PAMPs) recognized by host immune receptors like Toll-like receptor 5 (TLR5), triggering innate immune responses [41,42]. This interaction modulates immune evasion and influences bacterial persistence [43]. Adhesion is further reinforced by lectins LecA and LecB, which bind host glycoconjugates, enhancing biofilm stability and tissue colonization [44].

In clinical settings, particularly in CF airways, *P. aeruginosa* exploits its motility apparatus to invade tissues and form biofilms, creating protected niches resistant to host defenses and antibiotics [45]. The coordinated action of flagella and T4P enables bacterial aggregation into complex biofilm architectures, hallmarks of chronic infections. This adaptability, governed by environmental cues and signaling pathways, underscores the bacterium’s capacity to thrive in hostile host environments, contributing to its persistence in immunocompromised individuals [18,38].

### 2.4. Toxins and Enzymes Involved in Tissue Damage

*P. aeruginosa* secretes an array of potent toxins and enzymes, significantly contributing to its virulence and the pathogenesis of infections through tissue destruction and immune evasion [3]. A prominent virulence mechanism involves the secretion of exotoxins via the Type III secretion system (T3SS), which injects effector proteins directly into host cells. These exotoxins—ExoS, ExoT, ExoU, and ExoY—disrupt host cellular functions and promote immune evasion [46,47]. Specifically, ExoS and ExoT interfere with host cell signaling by modifying actin cytoskeleton dynamics by manipulating small GTPases such as Rho, Rac, and Cdc42. This interaction results in the collapse of actin stress fibers, breakdown of intercellular junctions, loss of tissue integrity, and increased bacterial invasiveness [47,48]. ExoU exhibits phospholipase activity, inducing rapid membrane damage, host cell lysis, and significant lung injury. Strains expressing ExoU are associated with hypervirulence, leading to severe infections and treatment complications [49,50]. ExoY causes the accumulation of cyclic nucleotides, destabilizing the cytoskeletal structure and thus further impairing cellular integrity and function [51].

*P. aeruginosa* also produces other significant toxins and enzymes through various secretion pathways. Elastases (LasA and LasB), secreted primarily through the Type II secretion system (T2SS), degrade extracellular matrix components and host cell junctions, facilitating bacterial spread within tissues [52,53]. Alkaline protease (AprA), protease IV (PrpL), and phospholipase C actively degrade host proteins, including immunoglobulins and surfactants, leading to extensive tissue damage and promoting immune evasion [54]. Exotoxin A (ExoA), released via T2SS, also significantly contributes to host cell apoptosis and immune disruption [55].

The production of pyocyanin, a phenazine pigment, induces oxidative stress by generating reactive oxygen species (ROS) [34]. These ROS damage host tissues and suppress immune cell function, exacerbating infection severity [46]. Furthermore, hydrogen cyanide (HCN), another toxic metabolite, contributes to oxidative stress and tissue injury [39,56]. Rhamnolipids, surfactant-like virulence factors essential for biofilm formation, play a dual role in biofilm stability and destruction of host tissues. Their presence enhances bacterial resistance to immune responses and antimicrobial agents, compounding infection control challenges [57,58].

Exolysin (ExlA), secreted via the Two-Partner Secretion system (TPS), forms pores in host cell membranes, triggering inflammasome activation, pyroptosis, and tissue disruption via ADAM10-mediated cleavage of cadherins, further compromising cellular integrity [59,60]. Additionally, the Type VI secretion system (T6SS) mediates bacterial competition and modulates host-pathogen interactions, thus influencing infection dynamics and persistence [19,35].

### 2.5. Immune Evasion Strategies

*P. aeruginosa* employs diverse strategies to evade host immune responses, enabling persistent infection and resistance to antimicrobial treatment. A central component of its immune evasion involves manipulating lipopolysaccharides (LPS), critical elements of its outer membrane. LPS triggers host inflammatory responses via interactions with receptors such as Toll-like receptor 4 (TLR4); however, modifications in LPS structure help the bacterium evade TLR recognition, reducing immune activation and facilitating persistent colonization [61,62].

Additionally, *P. aeruginosa* leverages secretion systems, particularly the T3SS and T6SS secretion systems, to deliver virulence factors directly into host cells. These effectors manipulate immune responses, promote bacterial survival, and assist in immune evasion [63]. Toxins such as ExoA disrupt host cell protein synthesis, further impairing immune responses. Elastases (LasA, LasB) and various proteases degrade host immune mediators, weakening the host’s defensive mechanisms and enhancing bacterial dissemination [33,52,60].

Biofilm formation constitutes a crucial immune evasion tactic, protecting bacterial communities within a self-produced matrix. This biofilm matrix shields bacteria from immune cell detection and antibiotic penetration [27]. Alginate production, especially significant in pulmonary infections, additionally prevents recognition by immune cells and enhances persistence within the host environment [64]. Surface-bound factors, such as pili, flagella, and LPS, promote adherence to host tissues, assist initial infection stages, and mask bacteria from immune surveillance, further aiding chronic colonization [65].

To counter host-generated oxidative stress, *P. aeruginosa* produces reactive ROS-scavenging enzymes, including catalases and peroxidases, regulated by transcription factors SoxR and OxyR. These enzymes neutralize oxidative attacks from immune cells, thus ensuring bacterial survival in hostile environments [66,67].

Efflux pumps, notably MexAB-OprM, significantly contribute to immune evasion by actively expelling antibiotics, enhancing intrinsic resistance, particularly among MDR and extensively drug-resistant (XDR) strains [50,68].

Furthermore, extracellular vesicles (EVs), such as outer membrane vesicles (OMVs), outer-inner membrane vesicles (OIMVs), and explosive outer membrane vesicles (EOMVs), transport virulence factors and antibiotic resistance enzymes (e.g., β-lactamases, aminoglycoside-modifying enzymes), facilitating further immune evasion and antibiotic resistance [69,70,71]. eDNA, released through bacterial autolysis, fortifies biofilm structure, enhancing resistance to immune attacks and antibiotic treatment [72,73].

### 2.6. Antibiotic Resistance

Antibiotic resistance significantly contributes to the virulence of *P. aeruginosa*, complicating therapeutic interventions and exacerbating clinical outcomes [74]. This resistance arises from intrinsic and acquired mechanisms, presenting notable challenges in managing infections.

Intrinsic resistance in *P. aeruginosa* involves several mechanisms, notably the reduced permeability of its outer membrane, modifications of outer membrane proteins (e.g., OprI and OprL), and the active expulsion of antibiotics via efflux pumps such as MexAB-OprM [2,75]. These efflux pumps effectively remove antibiotics and other toxic substances from bacterial cells, elevating the organism’s resistance profile [76].

Biofilm formation significantly enhances antibiotic resistance, complicating therapeutic interventions. Within biofilms, bacterial cells exhibit increased resistance to antibiotic penetration and evade immune detection, making infections more persistent and difficult to treat. This biofilm-associated resistance underscores the clinical challenge of eradicating *P. aeruginosa* infections, particularly in chronic cases [8,28].

Moreover, *P. aeruginosa* expresses a variety of antibiotic-degrading enzymes, notably β-lactamases, which neutralize key classes of antibiotics, including penicillins and cephalosporins [77]. The acquisition and horizontal transfer of resistance genes further enhances this resistance, frequently mediated by extracellular vesicles, including OMVs, OIMVs, and EOMVs. These extracellular vesicles facilitate the horizontal transfer of resistance determinants between bacterial cells, significantly accelerating the spread of resistance genes within bacterial populations [78,79].

## 3. Inhibitors of QS in *P. aeruginosa*

Strategies to quench QS in *P. aeruginosa* focus on disrupting key stages of its communication system to reduce pathogenicity. One approach targets the synthesis of AIs through enzymes like lactonases and acylases, which degrade or modify AIs, preventing them from activating QS receptors. Another strategy uses small molecules or structural analogs that bind to QS receptors, blocking AI binding and gene activation. Additionally, antibodies and scavenger proteins can sequester QS molecules. These QSIs can complement antibiotics by preventing biofilm formation and virulence factor production, enhancing bacterial susceptibility to the host immune system, and reducing resistance [80,81]. QSIs, as shown in Figure 1, highlight the diverse strategies targeting specific components of the QS pathway in *P. aeruginosa.*

### 3.1. Plant-Derived Natural Inhibitors of QS

Plant-derived compounds employ multifaceted strategies to disrupt *P. aeruginosa* QS, primarily interfering with acyl-homoserine lactone (AHLs) synthesis, receptor binding, and virulence gene expression (Table 1) [82]. Extracts from *Psidium guajava* leaves suppress LasR, RhlR, and CviR receptors via beta-caryophyllene and nerolidol, reducing pyocyanin, pyoverdin, and biofilm formation [83]. Similarly, *Hypericum perforatum* extracts inhibit LasI/R (71.33%) and RhlI/R (57.41%) systems, as demonstrated by diminished Green Fluorescent Protein (GFP) expression in *lasB*-GFP and *rhlA-*GFP reporter strains [84]. Molecular docking studies reveal that chlorogenic acid in *Prunus avium* stalks binds LasR, RhlR, and PqsR, reducing swarming motility (86%) and biofilm formation (75%) [85]. At the same time, *Nepeta curviflora* methanolic extract attenuates AHL secretion and downregulates biofilm-associated *pslA* and *pelA* genes [86]. Polyphenols like caffeoylmalic acid from *Salix tetrasperma* form hydrogen bonds with LasR and PqsR, impairing protease activity and motility [87].

Direct suppression of QS regulators is a recurring mechanism. *Musa acuminata* peel extract and its metabolite 5-hydroxymethylfurfural downregulate *lasI*, *lasR*, *rhlI*, and *rhlR*, reducing biofilm proteins and exopolysaccharides [88]. Green coffee extract at 2.5 mg/mL decreases *lasI* and *lasR* expression via chlorogenic acid [89]. *Diphysa americana* and *Hibiscus sabdariffa* inhibit swarming and ExoU toxin secretion, correlating with reduced murine pathogenicity [90]. European herbs like *Fragaria vesca* and *Matricaria chamomilla* disrupt AI-1/AI-2 signaling, suppressing biofilm and virulence [91], while ginseng (*Panax* spp.) impairs motility genes, enhancing bacterial clearance [92].

Biofilm and motility disruption are critical for reducing pathogenicity. *Syzygium cumini* ethyl acetate fraction inhibits biofilm by 86% via RhlG/NADP and LasR interactions [93]. *Teucrium polium* reduces swarming (23.66%) and swimming (35.25%) without affecting AHL production [94], whereas *Persicaria maculosa* and *Bistorta officinalis* abolish swarming at 50 μg/mL by inhibiting LasR [95]. *Syzygium aromaticum* and *Eucalyptus camaldulensis* competitively inhibit QS receptors, reducing biofilm [96]. Ginger and wild blueberry extracts downregulate *ndvB* and *pelC* by 10.4-fold, dispersing biofilms [97].

Synergy with antibiotics enhances therapeutic efficacy. *Fuzheng Touxie Jiedu Huayu* decoction reduces ceftazidime minimum inhibitory concentration (MIC) from 128 to 64 μg/mL by inhibiting MexAB-OprM efflux pumps and QS genes (*lasI*, *rhlR*) via quercetin and baicalein [98]. Panchvalkal formulation induces nitrosative stress, downregulating *norB* and *nirS* to disrupt the biofilm and QS [99] while altering oxidative stress and iron homeostasis genes [100]. LasR, a central QS regulator, is inhibited by boeravinone O (*Boerhavia diffusa*) and daidzein dimethyl ether (*Albizzia lebbeck*). Boeravinone O binds LasR’s ligand-binding domain with higher affinity than native autoinducers (3-oxo-C12-HSL/C4-HSL), destabilizing its structure and blocking virulence activation [101]. Similarly, daidzein dimethyl ether suppresses *lasR* and downstream genes (*lasB*), reducing biofilm, elastase, and pyocyanin by >50% [102]. Flavonoids like Mosloflavone bind LasR/RhlR, downregulating *lasI*, *lasR*, *rhlI*, and *rhlR* [103], while wogonin (*Agrimonia pilosa*) suppresses *pqsA, pqsR*, and *lasB*, impairing exopolysaccharides (*Psl*) and motility [104].

Luteolin modulates host immune responses by reducing TNF-α, IL-1β, and enhancing antioxidant genes (*sod, cat*) [105]. Quercetin and allicin inhibit biofilm-associated EPS, reducing proteins, carbohydrates, and eDNA [106]. Pinocembrin and vestitol target SagS in MDR strains, reducing biofilm and resistance [107]. Essential oils (EOs) from *Cuminum cyminum* and *Carum carvi* inhibit QS via cuminaldehyde and carvone, blocking LasR/CviR [108]. *Cinnamomum camphora* EO reduces violacein (63%) and biofilm (77.64%) by downregulating *cviI, cviR*, and *vioA-E* via linalool and eucalyptol [109]. Sunflower EOs (Agsun cultivars) bind LasR with high affinity (−66.42 kcal/mol), displacing 3OC12-HSL [110]. *Zingiber cassumunar* EOs synergize with tetracycline (FICI = 0.5), reducing pyocyanin and protease activity [111].

Berberine inhibits PqsA, disrupting alkylquinolone signaling and pyocyanin production [112]. Caffeine mimics 3-oxo-C12-HSL, blocking LasR activation and suppressing swarming [113]. Trigonelline hydrochloride targets Las/Rhl/Pqs systems, altering biofilm architecture [114]. Compound **C25** (*Plumula nelumbinis*) binds LasR/PqsR, silencing QS cascades [115]. Ricinine derivative 7 destabilizes AHL signaling, reducing biofilm [116]. Bakuchiol, a monoterpenoid from *Cullen corylifolium*, selectively inhibits the *pqs* system by binding PqsR, destabilizing its structure through interactions with the ALA-168 residue. This reduces *pqsA* expression by 70%, suppressing pyocyanin (65%), hydrogen cyanide (40%), elastase (55%), lectin (50%), and bacterial motility (>60%) while enhancing survival in *Caenorhabditis elegans* and *Brassica pekinensis* models by 80% [117]. Carvacrol exhibits broader anti-QS activity by targeting LasI (−5.932 kcal/mol), LasR (−7.469 kcal/mol), and BswR (−4.42 kcal/mol) via hydrogen bonds at critical residues (Ile107, Tyr47, Leu57), reducing C12-AHL levels by 60% and downregulating *lasR* by 50% [118,119].

Isoeugenol (400 μM) inhibits QS-regulated pyocyanin, rhamnolipids, exopolysaccharides (Psl), and biofilms (>70%) by binding LasI, LasR, PqsE, and SdiA (−4.8 to −6.4 kcal/mol). At the same time, isovanillin suppresses *las* and *pqs* systems at sub-MICs, enhancing antibiotic susceptibility via destabilized QS-mediated resistance [120,121]. Paeonol downregulates *lasI/R*, *rhlI/R*, and *pqsA/R* genes, reduces AHL signaling, and disrupts biofilm architecture [122]. Vanillin specifically inhibits PqsR (−7.3 kcal/mol), suppressing QS signaling and synergizing with colistin to improve *G. elegans* survival [123], whereas ortho-vanillin blocks RhlR activation by C4-HSL, preserving LasR functionality [124].

Phillyrin (0.25 mg/mL) suppresses pyocyanin, rhamnolipids, and elastase without bactericidal effects, reducing biofilm formation by 84.48% [125]. Sesamin and sesamolin (75 μg/mL) inhibit Las and Rhl systems, downregulating *lasI*, *rhlI*, and virulence genes (*phzM, rhlA*), enhancing *C. elegans* survival [126]. 6-Gingerol broadly targets LasR, RhlR, QscR, and PqsR, reducing biofilm formation and virulence factors by 40–70% [127]. Curcumin (12.5–50 μM) and baicalein (500 μM) inhibit pyocyanin and motility but lack biofilm efficacy, highlighting variable phenolic QSI potency [128].

Farnesol and tyrosol transcriptionally inhibit *lasI* and *rhlI*, disrupting hierarchical QS [129]. Thymoquinone (0.5–2 mg/mL) suppresses Las, Rhl, and QS systems, reducing pyocyanin (~73%), proteases (~70%), and biofilms (~63%) via gene downregulation (*lasI/R*, *pqsA-E/H*) and receptor binding (LasR, RhlR) [130,131]. Psoralen binds LasR, RhlR, and PqsR, reducing proteases and motility and enhancing *C. elegans* survival by 70% [132]. Umbelliferone downregulates *rhlR*, *lasA*, and *algL*, reducing pyocyanin (55%) and biofilm biomass (50%) while quadrupling ciprofloxacin susceptibility [133]. The synthetic coumarin 4t inhibits biofilms (IC50: 3.6 μM) and chelates iron, destabilizing pyoverdine and enhancing tobramycin efficacy [134].

Receptor antagonists like chlorogenic acid, methyl gallate, and falcarindiol competitively bind LasR/RhlR/PqsR, suppressing virulence [102,135,136]. Mangiferin derivatives (Zinc D/E) outperform azithromycin in biofilm inhibition [137]. Transcriptional modulators such as malonate and pyranoanthocyanins shift QS gene expression, while lutein and sennoside A suppress *las/rhl/pqs* genes [138,139,140,141]. Astragaloside IV and eugenol inhibit efflux pumps, limiting autoinducer release [142].

QS signal degradation strategies include malonate-induced lactonolysis, CS-g-CA-mediated AHL/c-di-GMP reduction, and coumarin-chalcone C9 suppression of 3-oxo-C12-HSL, C4-HSL, and PQS [138,143]. Biofilm disruption via EPS reduction (1,8-cineole), oxidative stress (CS-g-CA), and swarming inhibition (cycloartane triterpenes) further attenuates pathogenicity [144,145].

Synergy with antibiotics is exemplified by DDAG-azithromycin/gentamicin combinations, cinnamoyl hydroxamates enhancing gentamicin dispersal, and compound **2i** improving *G. mellonella* survival with ciprofloxacin/tobramycin [146,147,148]. In vivo efficacy is demonstrated by falcarinol in burned mice and compound **5f** in zebrafish [98,149].

### 3.2. Nanoparticles as Inhibitors of QS

Nanoparticles (NPs) represent a promising class of QSIs capable of disrupting bacterial communication pathways without promoting antimicrobial resistance (Table 2) [150].

Silver nanoparticles (AgNPs) exhibit multifaceted anti-QS mechanisms, with propolis-synthesized AgNPs reducing AHL-dependent violacein production in *Chromobacterium violaceum* by 75% through AHL suppression [151]. Multimodal Ag-chitosan-acylase nanoparticles (AgCS@AC) combine acylase I-mediated AHL degradation with biofilm eradication, achieving a 55% reduction in *P. aeruginosa* biofilms [152]. *Eruca sativa*-derived AgNPs further demonstrate efficacy by suppressing pyocyanin (68.8%) and exopolysaccharide (57.6%) production via inhibition of the *las* and *rhl* systems [153]. Proteomic analyses corroborate these effects, revealing AgNP-induced downregulation of QS genes (*lasI, rhlI*) and virulence factors (elastase, rhamnolipids) alongside oxidative stress-mediated biofilm destabilization [154]. However, subinhibitory AgNP concentrations paradoxically enhance bacterial motility and biofilm formation in certain strains, emphasizing the critical role of dosage optimization [155].

Zinc oxide nanoparticles (ZnO-NPs) inhibit QS by repressing *lasI* transcription and AHL synthesis [156]. Nanospiked ZnO structures impair biofilm attachment by suppressing protease and exopolysaccharide production [157]. At the same time, curcumin-loaded ZnO nanocomposites synergize pH-dependent curcumin release and ROS generation to inhibit *lasR* and *rhlR*, reducing pyocyanin and rhamnolipid levels [158]. Selenium nanoparticles (SeNPs) exhibit dual mechanisms, docking into LasI and RhlR proteins to block AHL synthesis and biofilm gene expression [159]. SeNPs achieve 80% inhibition of violacein in *C. violaceum*, whereas tellurium nanoparticles (TeNPs) disrupt signal perception at lower concentrations [160].

Titanium dioxide nanoparticles (TDN) enhance antibiotic efficacy by downregulating *lasI, rhlR*, and efflux pump genes [161]. Cerium dioxide nanoparticles (CeO_2_-NPs) mimic haloperoxidases, generating halogenated compounds that reduce biofilms by 85% [162]. Functionalized NPs, such as eugenol-coated gold (Eugenol_Au) and Fe_3_O_4_@EUG, suppress virulence factors through ROS generation and QS gene modulation [163,164]. *Salmonella enterica* co-loaded with eugenol and ofloxacin synergistically disrupts biofilms [15], while alginate-encapsulated meta-bromo-thiolactone (mBTL) inhibits LasR/RhlR, reducing pyocyanin in a dose-dependent manner [165].

Ulusoy et al. demonstrated that Zeolite 4A adsorbs 3-oxo-C12-HSL, reducing *P. aeruginosa* biofilms (87%) and pyocyanin (98%) [166]. In the same way, Yttrium oxide nanospheres inhibit AHL signaling and initial biofilm adhesion [167], and copper nanoparticles (CuNPs) destabilize biofilms via membrane damage and QS gene repression [168]. In contrast, the metallopharmaceutical Sivanar Amirtham shows ambiguous QS-modulating effects, warranting further mechanistic investigation [169].

### 3.3. Enzymes, Peptides, and Proteins as Inhibitors of QS

QS-quenching enzymes disrupt *P. aeruginosa* virulence by degrading AHLs, the signaling molecules essential for QS (Table 3). For example, YtnP, an N-acyl-homoserine lactonase from *Bacillus velezensis*, hydrolyzes AHLs, significantly reducing EPS synthesis and biofilm formation [170]. Other lactonases, including AiiM, AidB, MomL, and AiiA KMMI17, inactivate AHLs via lactone ring cleavage, suppressing pyocyanin, elastase, and biofilm production without inhibiting bacterial growth [171,172,173]. These enzymes exhibit broad substrate specificity against various AHLs (e.g., C4-HSL, 3OC12-HSL), disrupting the *las, rhl*, and *pqs* QS systems. Their efficacy in biofilm control is demonstrated in simulated water systems and infection models, underscoring their therapeutic potential [174,175].

QS receptors, such as LasR and RhlR, are direct targets for inhibition. The host defense peptide CRAMP synergizes with colistin to downregulate *lasR, lasI, rhlR*, and *rhlI*, reducing pyocyanin (32%) and rhamnolipid (40.5%) synthesis while destabilizing biofilms [176]. Synthetic peptides (WSF, FASK, YDVD) bind the ligand-binding domain of LasR, inhibiting biofilm-associated genes (*algC, pslA, pelA*) at subinhibitory concentrations [177]. Phage-derived proteins PIT2 and Aqs1 also interfere with LasR: PIT2 disrupts LasR-mediated transcription, suppressing elastase and biofilm formation [178], whereas Aqs1 obstructs LasR-DNA-binding, attenuating pyocyanin and motility [179].

Cyclic dipeptides, such as cyclo(L-Pro-L-Tyr), competitively bind LasR with an affinity comparable to 3OC12-HSL, downregulating *lasI* and *rhlI* [180]. Antimicrobial peptides like G3/C8G2 induce conformational changes in LasR, reducing virulence factor expression and biofilm biomass by >95% [180]. Nesfactin, a lipopeptide, combines AHL degradation with receptor binding, while the deep learning-optimized peptide PA-Win2 suppresses *las*, *rhl*, and *pqs* genes, degrading pre-formed biofilms in MDR strains [181,182].

Dual-function agents merge QS inhibition with bactericidal activity. The dendritic peptide G3KL permeabilizes bacterial membranes, causing cytoplasmic leakage, while M59 inhibits the MvfR regulator, reducing pyocyanin [183]. A β-peptide polymer (20:80-Bu: DM) disrupts membranes and suppresses QS genes (*las, rhl, pqs*), eradicating biofilms and outperforming levofloxacin in murine infection models [184]. These dual mechanisms enhance therapeutic efficacy by simultaneously targeting virulence and viability.

Biofilm matrix disruption indirectly impairs QS. Micafungin, a lipopeptide, reduces alginate and glucan synthesis, destabilizing biofilm architecture and enhancing antibiotic penetration [185]. Recombinant lectin rHPLOE binds biofilm-associated rhamnose, dispersing mature biofilms and suppressing di-rhamnolipid production, a QS-regulated biofilm component [186].

QSI universally attenuates virulence factors, including pyocyanin, elastase, and proteases. For instance, AiiM lactonase reduces elastase activity by 60% [171], while Olivancillaria-derived peptides inhibit pyoverdine by 72% [187]. Biofilm biomass is reduced by >90% using CRAMP/colistin combinations [176] and β-peptide polymers [184]. These effects translate to improved in vivo outcomes: PIT2 enhances survival in *C. elegans* and HeLa cell infection models [178], while PA-Win2 mitigates pneumonia and catheter-associated infections [182].

### 3.4. Synthetic and Derived Molecular Inhibitors

Synthetic and derived molecular inhibitors targeting *P. aeruginosa* employ diverse mechanisms to disrupt QS, efflux pumps, and virulence pathways, offering promising strategies to counter antibiotic resistance (Table 4) [188].

Diclofenac sodium exemplifies dual functionality by suppressing QS and inhibiting efflux pumps via downregulating *mexB, mexX*, and *mexY* genes associated with MexAB-OprM and MexXY-OprM systems. However, compensatory upregulation of *mexA* and *oprM* is observed. This inhibition enhances gentamicin’s efficacy by restoring antibiotic susceptibility [189,190]. Similarly, ketone- and cyano-selenoesters impair efflux pump activity, improving antimicrobial retention while disrupting QS through interference with autoinducer-1 (AI-1) and autoinducer-2 (AI-2) signaling. Competitive inhibitors like 5-hydroxymethylfurfural (5-HMF) bind LasR and RhlR receptors, key regulators of the Las and Rhl QS circuits, reducing pyocyanin, protease, elastase, and chitinase production by 40–85% and impairing biofilm formation and motility at subinhibitory concentrations [191]. Azithromycin suppresses AHL synthesis, diminishing QS-mediated virulence factors such as pyocyanin and biofilms. At the same time, phenylalanine-arginine β-naphthylamide (PAβN) inhibits efflux pumps involved in QS signal export, albeit with less potency than AZM [192]. High-throughput screening has identified 30 novel PqsR antagonists that competitively bind the PQS site, blocking *pqsE* activation and disrupting phenazine biosynthesis without affecting bacterial growth [193]. Cephalosporins such as cefepime and ceftazidime inhibit LasR, PqsR, and CviR receptors at sub-MIC levels, reducing pyocyanin, motility, and biofilm formation [194]. Compound Y-31, derived from the AOZ-1 scaffold, interacts with QS regulatory proteins, reducing pyocyanin (22.48%), elastase (22.67%), and biofilms (40.44%). At the same time, lamivudine (LAM) docks into LasR, RhlR, and PqsR, downregulating *lasI* and *pqsR* by 82.9% and 91.2%, respectively, and protecting mice from lethal infections [195,196]. Macrolides like erythromycin downregulate *lasI, lasR, rhlI*, and *pqsA* by 65.5–81.3%, synergizing with meropenem to enhance growth inhibition [197,198]. Furazolidone reduces *lasR, rhlR*, and *pqsR* expression, impairing biofilms, motility, and proteases [199,200]. At the same time, FDA-approved drugs such as erythromycin, chloroquine, and levamisole suppress *lasI* and *rhlI*, diminishing virulence in murine sepsis models [201,202].

Efflux pump inhibitors and siderophore disruptors further complement QS-targeted strategies. Nitrofurazone and erythromycin estolate inhibit PqsE, suppressing pyocyanin, rhamnolipids, and biofilms while relieving operon repression to enhance tobramycin efficacy [201]. The siderophore inhibitor HMMN disrupts iron acquisition and QS, reducing siderophores (69.37%), biofilms (28.24%), and pyocyanin (36.06%) via downregulation of *lasI, rhlR, mvfR*, and *pchG* [203]. Collectively, these inhibitors attenuate virulence traits, with pyocyanin production reduced by 22.48–36.06%, biofilm formation impaired by 28–45%, and motility disrupted via cephalosporins and furazolidone [194,199]. Protease activity declines by 40% with LAM and furazolidone. Synergies with antibiotics are notable: erythromycin potentiates meropenem, while nitrofurazone enhances tobramycin, suggesting combinatorial therapies to counter resistance [198,201]. In vivo, efficacy is demonstrated by Y-31 extending *C. elegans* survival, LAM protecting mice, and repurposed FDA drugs improving survival in murine sepsis [195,196,202].

The hierarchical Las, Rhl, and Pqs QS systems are prime targets for antivirulence strategies. Las inhibitors like niclosamide reduce 3OC12-HSL production, though efficacy varies across isolates, while clofoctol competitively targets PqsR to suppress PQS synthesis with broader consistency [204]. Furanone C-30 disrupts LasR folding, impairing QS-regulated gene expression and swarming motility [205], and brominated furanone GBr suppresses pyocyanin, biofilms, and the Type III secretion system (T3SS), attenuating murine infections [206]. N-alkylimidazoles, particularly octylimidazole, inhibit pyoverdine and biofilms via LasR interaction [207], while ibuprofen selectively inhibits Rhl by reducing C4-HSL synthesis [208]. Non-competitive inhibitors MHY1383 and MHY1387 suppress LasR and RhlR at picomolar concentrations, lowering c-di-GMP levels and improving survival in infection models [209]. RhlR antagonists like alkynyl ketone compound **30** block BHL binding, inhibiting rhamnolipids and biofilms [210], and benzimidazolium salts reduce elastase and pyocyanin by up to 64% [85]. Dimetridazole with ribavirin broadly targets LasR, RhlR, and PqsR to downregulate QS genes [211], while furazolidone suppresses *lasR, rhlR*, and *pqsR* expression, alleviating murine lung injury [200].

Pqs system inhibitors include thioether-linked cefoperazone Ce-2-ones, which competitively block PqsR to reduce alkylquinolone signaling [212], and quinazolinone derivatives like compound **61**, which bind PqsR with nanomolar affinity, suppressing PQS and HHQ production [213]. Thiazole-containing quinazolinones (compounds **18** and **19**) inhibit PqsR at sub-300 nM IC50 [214], and quinoline derivative compound **1** competitively antagonizes PqsR, reducing pyocyanin and elastase [215]. Thiazole-based inverse agonists (compounds **27** and **34**) suppress PqsR basal activity [216], while multi-target inhibitors like 4-fluorophenyl-5-methylene-2(5H)-furanone 23e inhibit Las, Rhl, and Pqs, enhancing ciprofloxacin activity [217]. Hybrid N-acylcysteines disrupt LasR and biofilms by downregulating pslA and lasI [218] and thiazolo-indolin-2-ones dual-target QS and dihydrofolate reductase [219].

Biofilm-specific strategies include QSI-polymer conjugates (e.g., P2-QSI), which enhance biofilm penetration and ciprofloxacin activity by 70% [220], and L-HSL, which disrupts EPS and motility [221]. 3-Hydroxypyridin-4(1H)-one hybrid inhibits Las and Pqs systems, increasing antibiotic susceptibility [222], while 1,3-diaminopropane suppresses flagellin synthesis and *lasR* expression [223]. Iron chelation synergizes with QS inhibition; compound **10d** binds FpvA, disrupting pyoverdine-mediated iron uptake [224]. Repurposed drugs like cefoperazone inhibit PQS synthesis, synergizing with ciprofloxacin [225], and virtual screening identifies quinazolinones and quinolones as optimized PqsR inhibitors [226]. Resistance to Las-targeting inhibitors is more prevalent than for Pqs-targeting compounds, highlighting the latter’s therapeutic potential [204]. Non-antibiotic approaches, such as efflux pump inducers Cou-1 and Cou-2, reduce T3SS exotoxins via GacS-GacA regulation [227].

Structural insights into inhibitor-receptor interactions have advanced rational design. Ketoprofen and G20 bind PqsR through hydrophobic and hydrogen bonds [228], while benzimidazole 6f engages critical PqsR residues [229]. Halogenated phenazines stabilize LasR’s allosteric site [230], and sulfoxide derivative 6b mediates LasR binding [231]. Repurposed adrenoreceptor blockers, including terazosin and doxazosin, stabilize QS receptors in inactive conformations, reducing biofilm formation and virulence in murine models [127,232]. β-Blockers like propranolol competitively bind LuxR-type receptors, downregulating QS genes [233]. Antidiabetic drugs metformin and vildagliptin synergistically reduce biofilm and virulence factors by binding LasR, RhlR, and PqsR [234], while sitagliptin suppresses motility and QS gene expression [235].

Allopurinol disrupts QS by binding LasR and RhlR, reducing biofilm formation by 61% [236], and cilostazol suppresses QS genes, synergizing with tobramycin [237]. Secnidazole blocks AHL receptor binding [238], and ostarine transcriptionally suppresses autoinducer synthesis [239]. Paracetamol inhibits biofilm formation via AbaR binding [240]. Synthetic derivatives like N-(2- and 3-pyridinyl)benzamides exhibit anti-QS activity [241], and ceftriaxone-silver complexes destabilize biofilms [242,243]. Hydrazide benzoxazole derivatives synergize with ciprofloxacin [244], and chalcone derivatives inhibit MvfR, reducing biofilms and pyoverdine [245]. Natural compounds like trans-cinnamaldehyde suppress *lasI* and *rhlR* expression [246], while benzaldehydes enhance antibiotic efficacy against biofilms [247].

Advanced delivery systems, such as nanoparticle-in-liposome platforms, co-deliver PqsR inverse agonists and antibiotics to biofilms, achieving > 90% reduction [248]. Multi-pathway targeting by Sodium New Houttuyfonate (SNH) and itaconimides disrupts QS and virulence [249,250]. Structural interference strategies include phosphate ester derivatives of chrysin and phenyloxadiazole sulfoxides [251,252]. Repurposed drugs like guanfacine block biofilm synthesis [253], and chromone-2-carboxamides exhibit high PqsR-binding affinity [254]. Dual-target inhibitors combining LasR antagonism with nitric oxide synthase inhibition impair virulence and survival in *C. elegans* [255].

### 3.5. Microorganisms Producing QSIs

Microorganisms employ diverse QSIs to disrupt *P. aeruginosa* communication through enzymatic degradation of signaling molecules, receptor antagonism, transcriptional repression, or modulation of secondary metabolic pathways (Table 5) [256].

A key mechanism involves enzymatic inactivation of AHLs, the primary QS signals in *P. aeruginosa*. Marine bacteria such as *Bacillus paralicheniformis* and *Vibrio alginolyticus* secrete lactonases and acylases that hydrolyze AHLs, suppressing biofilm formation by over 90% [257]. Similarly, *Bacillus cereus* and *Pseudomonas putida* degrade AHLs via the *aiiA* (lactonase) and *pvdQ/quiP* (acylase) genes, disrupting biofilm formation in MDR strains [258]. Streptomycetes like *Streptomyces griseoincarnatus* HK12 produce fatty acyl compounds that inhibit LasI, an AHL synthase, preventing signal synthesis entirely [259]. These enzymatic strategies block QS activation while preserving bacterial viability, reducing selective pressure for resistance.

Competitive antagonism of QS receptors represents another primary inhibitory strategy. Macrolactins from *Bacillus amyloliquefaciens* inhibit the PQS system, suppressing pyocyanin production [260]. At the same time, fungal metabolites such as chermesiterpenoids [261] and cladodionen [262] bind LasR and PqsR with higher affinity than native ligands, reducing elastase and rhamnolipid synthesis. Curvularin selectively antagonizes RhlR, blocking N-butanoyl homoserine lactone (BHL) binding and attenuating virulence in *C. elegans* infection models [263]. Similarly, the fungal compound tyrosol (*P. chrysogenum*) binds CviR homologs, suppressing pyocyanin by 63% [264]. These receptor-targeted QSIs prevent signal transduction, rendering *P. aeruginosa* less pathogenic without directly killing cells.

Transcriptional repression of QS-associated genes further disrupts virulence. Chitosan from shrimp shells reduces *lasR* and *rhlR* expression, lowering pyocyanin and biofilm production [265]. Actinomycin D from *Streptomyces cyaneochromogenes* suppresses *lasI, rhlI*, and *pqsR*, impairing AHL synthesis [266]. The NtrBC two-component system, activated by *Staphylococcus aureus* metabolites, interacts with QS pathways to upregulate pyoverdine and elastase; its deletion abolishes competitive virulence [267]. Gamma irradiation (1 kGy) synergizes with halophilic bacterial metabolites to repress *las/rhl* genes via oxidative stress, reducing biofilm biomass [268].

QSIs also target secondary signaling systems integral to virulence. Volatile organic compounds (VOCs) from *Spongiibacter nanhainus* downregulate iron uptake genes (*pchR, fpvA*), limiting intracellular Fe^2+^ and QS-dependent metabolism [269]. Chermesiterpenoids reduce c-di-GMP levels, destabilizing biofilm adherence [261], while exopolysaccharide EPS273 degrades eDNA, a critical biofilm matrix component [270]. Biosurfactants from *Lactiplantibacillus plantarum*, such as oleic acid, structurally mimic AHLs and reduce pyocyanin by 67% [271].

These strategies collectively suppress virulence factors: pyocyanin (23–95% reduction), elastase (35–57%), and rhamnolipids (34–98%) [272,273]. Biofilm formation is inhibited via the downregulation of *pslA, algP*, and *fleQ* [270,274], while pre-formed biofilms are dispersed by VOCs [269] and actinobacterial enzymes [275]. Non-bactericidal mechanisms reduce resistance risks; for example, *Delftia tsuruhatensis* C18-HSL disrupts QS without affecting growth [276].

QSIs enhance conventional antibiotics by sensitizing *P. aeruginosa*. Chitosan reduces ceftazidime MIC by 15-fold [265], while phthalate derivatives synergize with meropenem [277]. Probiotics like *L. plantarum* F-10 suppress motility and biofilm formation [278], and bacteriophages complement QSIs by lysing planktonic cells [279]. Marine-derived compounds, including lyngbyoic acid, exhibit low cytotoxicity, underscoring their therapeutic potential [280,281].

### 3.6. Other Inhibitors

Bromotyrosine derivatives from *Aplysinella rhax*—psammaplin A and bisaprasin—show strain-specific QS modulation. Bisaprasin broadly suppresses *lasB* (IC_50_: 3.53 μM) and *rhlA* (IC_50_: 2.41 μM) expression, while psammaplin A is more potent against *rhlA* (IC_50_: 4.99 μM vs. 14.02 μM for *lasB*). Both inhibit elastase production, with psammaplin A achieving more potent suppression (50–100 μM); molecular docking indicates its thiol monomer competitively binds LasR’s ligand-binding domain, disrupting QS. Notably, bisaprasin disrupts biofilm formation at ≥100 μM, unlike psammaplin A [282].

Palmitoleic acid (PMA) targets QS by reducing 3OC12-HSL and C4-HSL, dose-dependently suppressing pyocyanin production and downregulating *lasR, rhlR*, and *rhlI* [275]. Its altered membrane permeability lowers the intracellular-to-extracellular 3OC12-HSL ratio, delaying QS activation and reducing biofilm biomass.

Guanosine from *Melianthus comosus* extracts antagonizes QS by competitively binding LasR and CviR, with docking scores of –8.376 and –5.969 kcal/mol, respectively. It reduces biofilm formation (78.88% at the attachment phase and 34.85% during maturation), suppresses pyocyanin synthesis, and inhibits motility, with a MIC of 0.031 mg/mL [283].

Cyclodextrins, particularly α-CD, inhibit QS by sequestering hydrophobic autoinducers into inclusion complexes. At 10 mM, α-CD reduces pyocyanin by 58% (72 h) and pyoverdine by up to 70% (48 h) without affecting bacterial growth, functioning as a QS quencher and offering a strategy to circumvent antibiotic resistance [284].

### 3.7. Clustered Regularly Interspaced Short Palindromic Repeats-Based Inhibition of QS

The emergence of Clustered Regularly Interspaced Short Palindromic Repeats (CRISPR)-based genome-editing technologies has introduced novel strategies to disrupt QS pathways, offering a targeted approach to attenuate bacterial pathogenicity. By leveraging the precision of CRISPR systems to silence or disrupt QS-associated genes, researchers have demonstrated the potential to interfere with bacterial communication, thereby reducing virulence and biofilm-related resilience. This approach is auspicious in MDR pathogens, where traditional antibiotics often fail [285].

In *Escherichia coli*, CRISPR-Cas9 and CRISPR interference (CRISPRi) systems have successfully targeted QS-related genes such as *luxS*, *fimH*, and *bolA*. For instance, CRISPRi-mediated repression of *luxS*, a gene essential for autoinducer-2 (AI-2) synthesis, resulted in a 75–84% reduction in biofilm formation, alongside diminished adhesion and virulence [286]. Similarly, in *Salmonella enterica*, CRISPR-Cas9 targeting of *sdiA*, a receptor for exogenous AHLs, led to impaired biofilm formation and attenuated virulence in murine infection models [287]. The application of CRISPR-based strategies extends to *Vibrio* species, where Mobile-CRISPRi systems have been used to silence *luxR* and *luxO*, key regulators of bioluminescence and QS. Repression of these genes resulted in significant reductions in light production. It disrupted biofilm architecture, highlighting the potential of CRISPRi to modulate QS-regulated behaviors in environmentally and clinically relevant species [288]. Such findings in diverse bacterial models provide a foundational framework for adapting CRISPR technologies to *P. aeruginosa*, a notorious MDR pathogen whose QS systems are central to its virulence.

In *P. aeruginosa*, CRISPR-Cas9 and CRISPR-Cas3 systems have been deployed to target master regulators of QS, including *lasR* and *rhlR*. For example, CRISPR-Cas9-mediated disruption of *lasR*, a transcriptional activator of *N*-acyl-homoserine lactone (AHL) production, significantly reduced biofilm formation and pyocyanin synthesis, key virulence traits [289]. Similarly, the Type I-F CRISPR-Cas3 system was shown to degrade *lasR* mRNA, impairing QS signaling and attenuating virulence in both in vitro and murine infection models [290]. Computationally designed guide RNAs targeting the LasR gene, a key QS regulator, and constructed a corresponding vector for gene knockout. Experimental validation demonstrated that disrupting LasR expression significantly reduced QS activity and virulence factor production, aligning with the passage’s assertion that CRISPR systems can be adapted to disrupt bacterial communication networks [291]. These outcomes mirror the success observed in *E. coli* and *Salmonella*, where CRISPR-based QS inhibition led to diminished pathogenicity and biofilm resilience.

The convergence of evidence across bacterial species reinforces the viability of CRISPR-based QS inhibition as a therapeutic strategy for *P. aeruginosa*. The shared mechanistic principles—such as using guide RNAs to target conserved QS regulators and the resultant phenotypic attenuation—suggest that CRISPR systems can be broadly adapted to disrupt bacterial communication networks. For instance, the CRISPRi-mediated silencing of *lasR* in *P. aeruginosa* parallels the repression of *luxS* in *E. coli*, resulting in disrupted biofilm formation and reduced virulence [286,292]. Furthermore, the adaptability of CRISPR systems, exemplified by the use of dCas9 for reversible gene repression, offers flexibility in modulating QS without permanent genomic alterations, a feature critical for translational applications [293].

### 3.8. Comparative Analysis of QS Inhibitor Classes

QSIs span diverse chemical and biological classes, each with distinct mechanisms, efficacy profiles, and limitations. Microbial-derived inhibitors, such as macrolactins (e.g., amylomacrolactines A/B) and diketopiperazines (DKPs), disrupt QS by degrading AHLs or mimicking autoinducers. For instance, amylomacrolactines A/B suppress pyocyanin production [260], while DKPs from *B. cereus* reduce biofilm formation by 68.64% [294]. However, these compounds often exhibit narrow-spectrum activity and face scalability challenges due to complex biosynthesis pathways. In contrast, plant-derived phytochemicals like 6-gingerol and thymoquinone demonstrate broader QS inhibition by targeting multiple receptors (LasR, RhlR, and PqsR). Thymoquinone, for example, reduces pyocyanin by 73% at 2 mg/mL [131], while 6-gingerol suppresses biofilm formation by 42.74% [97]. Despite their potency, poor aqueous solubility and rapid metabolic degradation limit their therapeutic utility, though formulations like chitosan nanoparticles can enhance delivery [295] (Table 6).

Synthetic compounds, such as furanone C-30 and phenyloxadiazole sulfoxides, offer high specificity and tunability. Furanone C-30 binds LasR with a ΔG of −54.22 kcal/mol, reducing biofilm biomass by 50% [205], while compound **5b** (phenyloxadiazole sulfoxide derivative) inhibits LasB elastase with an IC_50_ of 8.7 μM [252]. Although these molecules often outperform natural analogs in potency (nanomolar to low micromolar IC_50_), issues like cytotoxicity (e.g., hemolysis at >100 μM) and resistance emergence hinder clinical translation [296,297]. Peptide-based inhibitors, including CRAMP and PA-Win2, act through dual mechanisms: CRAMP disrupts membrane integrity and downregulates *lasR*/*rhlI* genes, achieving 91.05% biofilm reduction at sub-MIC doses [176], while PA-Win2 eliminates biofilms by depolarizing bacterial membranes [182]. However, susceptibility to proteolytic degradation necessitates advanced formulations, such as hydrogels, to prolong stability [298].

Nanoparticles, particularly AgNPs and ZnO-NPs, combine QS inhibition with bactericidal effects. Propolis-synthesized AgNPs reduce violacein production by 75.24% [151], and ZnO-NPs inhibit *lasI* transcription by 70% [156]. Despite high efficacy (>85% biofilm inhibition), concerns over cytotoxicity and environmental persistence restrict their use [299]. Enzymatic inhibitors, such as AiiA lactonase and MomL, hydrolyze AHLs, reducing pyocyanin by >60% [172,173]. While enzymes provide precise targeting, their instability in vivo demands immobilization strategies (e.g., encapsulation) to maintain activity.

## 4. Molecular Mechanisms of Plant-Derived Compounds That Antagonize QS Receptors

### 4.1. Competitive Inhibition of LasR Through Ligand Mimicry

Numerous plant phenolics and terpenoids act as structural analogs of *P. aeruginosa*’s native autoinducers, competitively occupying the ligand-binding domain (LBD) of the LasR receptor. For example, 6-gingerol, eugenol, and naringenin insert into LasR’s AHL pocket via hydrogen bonding with residues such as Thr75, Asp73, and Tyr56, complemented by hydrophobic interactions with Ala127, Ile52, and Trp88. This steric blockade prevents 3-oxo-C12-HSL binding, as confirmed by molecular docking, GFP reporter assays, and thermal-shift analyses. The resulting inhibition suppresses *lasR* transcription, downstream QS regulons (*lasI*, *rhlI/R*), and virulence phenotypes like pyocyanin, elastase, and biofilm production [300,301]. Similar mechanisms are observed for zingerone, parthenolide, and catechin-7-xyloside, which mimic autoinducer geometry while forming π-stacking or hydrogen-bond networks with conserved LasR residues (e.g., Tyr64, Leu125). These interactions reduce LasR’s affinity for its cognate signal molecule by 10–100-fold, as quantified by microscale thermophoresis and dissociation constants [302,303,304].

### 4.2. Dual Targeting of QS Receptors and Synthases

Certain compounds disrupt QS by simultaneously binding LasR and LasI synthase, inhibiting signal perception and synthesis. Carvacrol docks into LasR’s LBD (ΔG = −7.47 kcal mol^−1^) through hydrogen bonds with Tyr47 and hydrophobic stacking, while also occupying LasI’s catalytic site via Zn^2+^ coordination and hydrogen bonding to Thr104/His399, reducing AHL production by >50%. Similarly, methyl gallate (MG) competitively engages LasR (Trp60, Arg61) and LasI (Arg30, Thr144), with ICM scores of −57.18 and −50.79 kcal mol^−1^, respectively. This dual interference lowers extracellular 3-oxo-C12-HSL levels and suppresses *lasR/I* transcription, as demonstrated by β-galactosidase assays and qRT-PCR. Eugenol-conjugated silver nanoparticles (Eu-AgNPs) extend this strategy by targeting LasR, LasI, and MvfR through hydrogen bonding (Tyr56, Ser129) and metal coordination, achieving >80% reductions in violacein, pyocyanin, and biofilm biomass [118,136,305].

### 4.3. Multi-Receptor Antagonism Across QS Hierarchies

Sesquiterpenoids (α-copaene, β-caryophyllene) and polyphenols (caffeoylmalic acid, epicatechin) exhibit broad-spectrum QS inhibition by targeting LasR, RhlR, and PqsR. Docking simulations reveal α-copaene forms π-alkyl interactions with Val76 (LasR) and Leu57 (CviR), while caffeoylmalic acid binds RhlR (ΔG = −74.39 kcal mol^−1^) via hydrogen bonds to Arg19/Asp42. These interactions suppress violacein synthesis in *C, violaceum*, and attenuate *P. aeruginosa* virulence outputs (e.g., 70% biofilm reduction, 83% pyocyanin decline). Similarly, zingerone and terpinen-4-ol competitively engage LasR, RhlR, and PqsR, reducing C4-AHL and PQS levels while downregulating *rhlI/R* and *pqsR*. Such multi-receptor targeting disrupts the interconnected Las-Rhl-PQS signaling network, amplifying antivirulence effects [83,87,306,307].

### 4.4. Enzymatic Interference with AHL Synthesis

Trans-cinnamaldehyde (CA) and reserpine inhibit LasI synthase, a key enzyme in AHL biosynthesis. CA occupies LasI’s S-adenosylmethionine cavity via π–π interactions with Phe27/Trp33, competitively blocking autoinducer synthesis. Reserpine further obstructs LasI’s catalytic channel through hydrogen bonds to Leu22/Ser109, reducing AHL levels by >70%. These mechanisms, validated by docking and GFP reporter assays, correlate with 10^2^–10^3^-fold repression of *lasI* and *rhlI* transcripts and diminished protease/rhamnolipid production [246,308].

### 4.5. Transcriptional Repression of QS Regulatory Genes

Subinhibitory concentrations of carvacrol, parthenolide, and *Acacia nilotica* polyphenols downregulate *lasR* and *rhlR* transcription by 57–90%, as quantified by qRT-PCR. This repression cascades into reduced expression of QS-controlled genes (*lasB*, *rhlA*), lowering elastase, pyocyanin, and biofilm biomass. Phenotypic assays confirm that transcriptional silencing, rather than bactericidal activity, drives virulence attenuation [118,309].

## 5. Mechanisms of Resistance to QSI in *P. aeruginosa*

Targeting QS with inhibitors has emerged as a promising antivirulence strategy, aiming to disarm the bacterium without directly killing it, thereby reducing selective pressure for resistance. However, the evolutionary adaptability of *P. aeruginosa* has led to the rapid development of diverse resistance mechanisms, undermining the efficacy of QSIs and complicating therapeutic outcomes [310]. These mechanisms range from genetic mutations in QS regulatory genes and compensatory activation of alternative signaling pathways to structural biofilm defenses and enzymatic degradation of inhibitors. Notably, the pathogen’s ability to overproduce signaling molecules, upregulate efflux pumps, and modify autoinducer structures further exemplifies its resilience [311]. Such adaptations are particularly problematic in chronic infections, such as those in CF patients, where biofilms and hypoxic microenvironments exacerbate resistance.

### 5.1. Mutations in QS Regulatory Genes

Genetic mutations in QS regulatory genes are a primary resistance mechanism, enabling *P. aeruginosa* to evade QSI by altering receptor function or signal transduction. Mutations in key QS regulatory genes, such as *lasR*, *rhlR*, and *pqsR*, are central to resistance against QSIs. *LasR* mutations, including point mutations, frameshift mutations, insertions, and deletions, frequently localize to the DNA-binding or ligand-binding domains, rendering the protein non-functional or constitutively active [312]. These mutations impair signal molecule binding (e.g., 3OC12-HSL) and transcriptional activation of virulence genes, leading to persistent biofilm formation and virulence factor production despite QSI exposure [313]. Similarly, *rhlR* and *pqsR* mutations disrupt ligand recognition (e.g., C4-HSL or PQS), causing signal-independent activation or reduced inhibitor efficacy [314]. Clinical isolates, particularly from chronic infections like CF, often harbor these mutations, confer adaptive advantages in hypoxic biofilm environments by activating alternative regulators like Anr [315]. Phenotypic consequences include sustained pathogenicity, reduced QSI susceptibility, and enhanced biofilm resilience [316].

### 5.2. Overproduction of QS Signal Molecules

*P. aeruginosa* bypasses QSI efficacy by overproducing signaling molecules, overwhelming inhibitor competition, and maintaining QS-mediated virulence. *P. aeruginosa* counteracts QSIs by overproducing signaling molecules such as AHLs and PQS [317]. Upregulation of biosynthetic enzymes (e.g., LasI, RhlI) elevates extracellular signal concentrations, saturating receptor sites, and overwhelming competitive inhibition [314]. Hyper-virulent strains leverage this mechanism to maintain virulence factor expression (e.g., pyocyanin, elastase) and biofilm formation [318]. Chemical analyses and reporter assays confirm elevated AHL levels in resistant strains, enabling persistent QS activity even under inhibitor pressure [319].

### 5.3. Activation of Alternative QS Pathways

When primary QS systems (e.g., LasI/LasR) are inhibited, *P. aeruginosa* activates compensatory pathways such as RhlI/RhlR and PqsR [314]. For example, *lasR* mutants exhibit upregulated *rhlR* and *pqsR* expression, enabling continued regulation of virulence genes and biofilm formation [320]. Cross-talk between systems ensures redundancy, allowing bacteria to bypass targeted QS inhibition [321]. Transcriptomic studies reveal pathway-switching dynamics, where inhibition of one system triggers overexpression of another, maintaining pathogenicity [322].

### 5.4. Efflux Pump Overexpression

Overexpression of efflux pumps, particularly MexAB-OprM, mediates QSI resistance by expelling inhibitors from bacterial cells [323]. Mutations in regulatory genes like *mexR* derepress pump expression, reducing intracellular QSI concentrations. This mechanism also confers cross-resistance to antibiotics, as MexAB-OprM exports diverse substrates [324]. Biofilm-associated strains further exploit efflux pumps, with hyperactive variants forming denser biofilms due to enhanced AHL export [325].

### 5.5. Catabolic Degradation of QSIs

Certain strains enzymatically degrade QSIs, such as furanone C30, via lactone ring hydrolysis. This catabolic activity neutralizes inhibitors, reducing their environmental efficacy. Prolonged QSI exposure selects for strains with enhanced degradative enzymes, posing challenges for long-term therapeutic use [326].

### 5.6. Biofilm-Mediated Resistance

Biofilms confer intrinsic resistance through physical and physiological barriers. The extracellular matrix (polysaccharides, eDNA) limits QSI penetration, while reduced metabolic activity in biofilm cells diminishes inhibitor susceptibility [327]. Biofilms also upregulate efflux pumps and facilitate horizontal gene transfer, accelerating resistance evolution [328].

### 5.7. Compensatory Activation of Virulence Pathways

QS-independent virulence pathways enable resistance by bypassing QS regulation. Mutants produce virulence factors (e.g., pyocyanin) via alternative signaling cascades, maintaining pathogenicity despite QSI application [329].

### 5.8. Structural Modifications of Autoinducers

Some strains modify autoinducer structures (e.g., altering AHL acyl chains or lactone rings) to evade QSI binding. These changes prevent inhibitor-receptor interactions, preserving QS signaling and virulence [330].

### 5.9. Environmental Modulation of QS Hierarchy

Environmental stressors (e.g., nutrient limitation, hypoxia) shift QS system dominance. Phosphate or iron scarcity upregulates RhlR or integrated quorum-sensing systems (*iqs*), enabling virulence factor production under suboptimal conditions [331].

### 5.10. Mutations Enhancing Autoinducer Affinity

Rare mutations in LuxR-like receptors increase autoinducer affinity, lowering activation thresholds. These gain-of-function mutations allow QS activation despite low signal concentrations, circumventing competitive inhibition [314].

## 6. Challenges and Limitations of QSIs

The emergence of antibiotic-resistant *P. aeruginosa* has driven interest in anti-QS therapies as innovative alternatives to conventional antibiotics. These agents aim to disrupt bacterial communication, attenuating virulence and reducing selective pressure for resistance. However, their clinical translation is hindered by multifaceted challenges spanning efficacy, safety, mechanistic understanding, and practical applicability, necessitating a critical synthesis of these limitations [332].

### 6.1. High Required Concentrations

The necessity for elevated concentrations to achieve therapeutic efficacy represents a pervasive challenge across multiple classes of QSI, undermining their clinical viability. Synthetic antimicrobial peptides (AMPs), such as WSF, FASK, and YDVD, exemplify this limitation, requiring doses as high as 1600 μg/mL to inhibit *P. aeruginosa* planktonic growth. These high concentrations raise concerns about cytotoxicity and complicate dosing regimens, as achieving such levels in vivo may exceed safe pharmacokinetic thresholds [177]. Similarly, natural product-derived inhibitors, such as ginger and wild blueberry extracts, demand impractical concentrations (5–25%) to achieve modest biofilm reduction. These levels are far beyond typical pharmacological dosing ranges, posing formulation challenges and risks of systemic toxicity due to the sheer volume or frequency of administration required [97].

Cyclodextrins further illustrate this issue while effectively disrupting QS signaling molecules like AHLs. Their antivirulence activity necessitates prolonged exposure (48–72 h) at 10–12.5 mM concentrations, rendering them unsuitable for acute infections or scenarios requiring rapid therapeutic action. Even synthetic small molecules, such as chromone-2-carboxamide derivatives, require 50 μM to inhibit 90% of biofilm formation—a concentration that may not be achievable in clinical settings due to solubility limitations or toxicity thresholds [254].

### 6.2. Cytotoxicity and Host Cell Damage

The non-selective activity of several QSI poses significant risks to host cells and tissues, limiting their therapeutic utility. Dendritic peptides, such as G3KL, exemplify this challenge through their membranolytic mechanism. While effectively disrupting bacterial membranes, G3KL indiscriminately targets eukaryotic cell membranes, leading to host cell lysis and tissue damage. This lack of selectivity arises from the peptide’s structural similarity to host defense peptides, compromising its ability to distinguish between pathogen and host cells [183]. Similarly, synthetic peptides like LIVRHK and LIVRRK, though potent QS suppressors, require high doses that exacerbate cytotoxicity, as seen in in vitro models of human epithelial cells [333].

Nanoparticle-based strategies introduce additional risks through oxidative stress. For instance, zinc-copper nanocomposites (ZnC-NCs) and eugenol-conjugated gold nanoparticles (Eugenol_Au NPs) generate ROS as part of their antimicrobial mechanism. While ROSs disrupt bacterial biofilms, they also induce oxidative damage in host tissues, impairing cellular repair mechanisms and exacerbating inflammation in chronic wounds or pulmonary infections [164]. AgNPs, though effective QSI, similarly risk accumulating in vital organs such as the liver and kidneys, where prolonged exposure can lead to organ dysfunction [334].

Natural product-derived inhibitors are not exempt from toxicity concerns. The venom-derived peptide PA-Win2, while effective at disrupting *P. aeruginosa* biofilm formation, exhibits off-target interactions with human neuronal and cardiovascular cells, necessitating extensive safety evaluations to mitigate risks of neurotoxicity or arrhythmias [182]. Mycotoxin-based compounds, such as citrinin, further illustrate this issue; even at subinhibitory concentrations, they disrupt host mitochondrial function, highlighting the delicate balance between antimicrobial activity and biocompatibility [272]. These examples underscore the critical need for selectivity profiling and structural modifications to minimize host-system collateral damage while retaining antivirulence efficacy.

### 6.3. Resistance Risks

The non-lethal nature of QS inhibition creates a fertile ground for bacterial adaptation, posing a significant threat to the long-term efficacy of antivirulence therapies. Subinhibitory concentrations of inhibitors, while sufficient to disrupt QS-mediated virulence, often fail to eradicate bacterial populations, enabling *P. aeruginosa* to develop phenotypic tolerance or genetic resistance [317]. For example, subtherapeutic doses of synthetic peptides like G3 and C8G2 exert selective pressure that favors the emergence of QS-deficient mutants or hypermutable strains, which bypass QS regulation to sustain virulence through alternative pathways [180]. Similarly, plant-derived extracts such as *N. curviflora* exhibit dose-dependent QS suppression. Still, at low concentrations, they inadvertently promote bacterial efflux pump activation or biofilm matrix remodeling, enhancing resilience against subsequent treatments [86].

Nanoparticles administered at sub-MICs further exacerbate resistance risks. Bio-synthesized AgNPs, while effectively reducing QS signal molecules, enhance bacterial motility and upregulate efflux systems like MexAB-OprM when used at sub-lethal doses. This adaptive response compromises the efficacy of AgNPs and may confer cross-resistance to conventional antibiotics [155]. Additionally, QSI-targeting single pathways, such as PqsR antagonists, leave Las and Rhl systems intact. Residual bacteria exploiting these redundant QS networks can regenerate virulent subpopulations, as observed in *P. aeruginosa* biofilms treated with high-throughput-screened PqsR inhibitors [193].

The persistence of resistant mutants is further amplified in chronic infections, where heterogeneous biofilms provide sanctuaries for QS-independent subpopulations. Enzymatic inhibitors like AHL-lactonases, which degrade signal molecules without killing bacteria, may inadvertently select for “cheater” strains that exploit public goods (e.g., proteases, toxins) produced by QS-active neighbors, perpetuating infection [171]. Combinatorial approaches—pairing QSI with antibiotics or phage therapy—are critical to mitigate these risks. For instance, coupling PqsR inhibitors with bactericidal agents like ciprofloxacin reduces bacterial load while suppressing virulence, minimizing opportunities for resistance evolution [244].

### 6.4. Pharmacokinetic and Formulation Challenges

The successful translation of QSI from bench to bedside is significantly hindered by instability, poor bioavailability, and complex delivery requirements. Peptide-based inhibitors, such as Nesfactin, exemplify these hurdles. While effective in vitro, Nesfactin degrades rapidly in physiological environments due to protease activity, necessitating hydrogel-based formulations to stabilize its structure and prolong its half-life. However, hydrogels introduce challenges, including variable drug release kinetics and limited tissue penetration in dense biofilms, which compromise therapeutic consistency [181]. Similarly, enzymatic inhibitors like MomL lactonase face delivery obstacles; their production relies on heterologous expression in *Bacillus brevis*, requiring purification and encapsulation for therapeutic use. This multi-step process escalates production costs and complicates direct administration, particularly systemic infections [335].

Nanoparticle formulations, though promising, grapple with instability in biological environments. ZnC-NCs, designed for pH-dependent release in acidic infection sites, often underperform in vivo due to physiological pH variability. For instance, in chronic wound models, fluctuating pH levels disrupt the controlled release of QSI, leading to subtherapeutic drug concentrations and inconsistent biofilm suppression [158]. Similarly, SLNs co-loaded with antibiotics and QSI face manufacturing complexities. While enhancing drug solubility, the lipid matrix is prone to destabilization during scale-up, resulting in batch variability. Chitosan coatings that improve mucosal adhesion further risk triggering immunogenic responses, limiting their use in immunocompromised patients [15].

Enzymatic QSI, such as AHL-lactonases from marine actinobacteria, suffer from poor in vivo stability. These enzymes are highly susceptible to proteolytic degradation in host tissues and lose activity at physiological temperatures or pH conditions [256]. For example, recombinant AHL-lactonase KMMI17, while effective in vitro, shows reduced activity in metal-deficient host environments, as its metallo-β-lactamase domain requires zinc ions for catalytic function—a resource often sequestered by the host during infection [172]. Natural product formulations face additional hurdles: chitosan, though effective in synergizing with antibiotics, exhibits erratic solubility in physiological fluids, leading to unpredictable bioavailability [265].

### 6.5. Specificity and Off-Target Effects

The lack of precision in targeting *P. aeruginosa* QS systems often results in unintended biological consequences, undermining both efficacy and safety. Enzymatic inhibitors, such as recombinant lectin rHPLOE, exemplify this limitation by binding exclusively to rhamnose-containing components of *P. aeruginosa* biofilms. While effective against strains producing rhamnolipid-rich matrices, this narrow specificity renders the enzyme useless against biofilms lacking these structures, significantly restricting its therapeutic scope [186]. Similarly, broad-spectrum enzymes like AidB degrade AHLs across bacterial species, indiscriminately disrupting QS communication in pathogenic and beneficial microbiota. This collateral damage risks dysbiosis, particularly in the gut or respiratory microbiomes, where commensal bacteria contribute to host defense and metabolic homeostasis [173].

Synthetic compounds further illustrate the trade-off between potency and specificity. Ketone-/cyano-selenoesters, designed to inhibit both AI-1 and AI-2 QS pathways, disrupt interspecies signaling networks in polymicrobial environments. While this broad activity may suppress virulence in *P. aeruginosa*, it destabilizes symbiotic microbial communities, potentially exacerbating infections by opportunistic pathogens [189]. Natural product-derived inhibitors, such as ortho-vanillin, selectively target the RhlR receptor but fail to inhibit Las or Pqs systems, allowing *P. aeruginosa* to maintain virulence through redundant QS pathways. This partial targeting is particularly problematic in chronic infections, where strain heterogeneity ensures the persistence of QS-active subpopulations [124].

Mechanistic ambiguity exacerbates specificity challenges. Cyclic dipeptides like tryptophan-containing CDPs interfere with QS in *P. aeruginosa* through poorly characterized interactions, raising concerns about off-target binding to host or microbial receptors unrelated to virulence [336]. Similarly, bromotyrosine-derived compounds like psammaplin A exhibit variable efficacy across QS-regulated genes (e.g., *lasB* vs. *rhlA*), suggesting inconsistent receptor affinity or unintended interactions with bacterial metabolic pathways [282]. These off-target effects complicate dose optimization, as higher concentrations may amplify unintended interactions.

### 6.6. Ecological and Safety Concerns

The environmental persistence and unintended biological interactions of QSI pose significant ecological and safety challenges, complicating their clinical and environmental deployment [337]. Nanoparticles, such as AgNPs and ZnO-NPs, exemplify these risks. While effective at disrupting QS signals and biofilms, AgNPs accumulate in aquatic ecosystems, persist indefinitely, and disrupt microbial communities. This environmental persistence promotes horizontal gene transfer of resistance markers among bacteria, exacerbating the global antibiotic resistance crisis [334]. Similarly, ZnO-NPs, though biodegradable under certain conditions, generate ROS that harm aquatic organisms and destabilize ecological balances in water systems [158].

Natural product-derived inhibitors introduce risks to both host microbiomes and human physiology. Fatty acyl compounds from *S. griseoincarnatus* non-specifically degrade QS molecules across bacterial species, disrupting beneficial commensal microbiota in niches like the gut or respiratory tract. Such dysbiosis can impair host immune function or metabolic health, creating vulnerabilities to secondary infections [259]. Phthalate derivatives, despite reported non-toxicity in initial studies, carry historical associations with endocrine disruption, raising concerns about chronic exposure effects such as hormonal imbalances or reproductive toxicity [277]. Mycotoxin-based compounds like citrinin further illustrate safety trade-offs; even at subinhibitory concentrations, they interfere with mitochondrial function in host cells, potentially exacerbating tissue damage in immunocompromised patients [272].

Non-specific therapeutic strategies amplify these risks. Though effective at generating ROS to disrupt QS, Gamma irradiation damages host tissues through oxidative stress, particularly in chronic wound or pulmonary infection models [268]. Similarly, phage-based QSI face regulatory hurdles due to their strain-specific activity and potential to provoke immune reactions, which may worsen inflammatory responses in sensitive populations [279]. Plant-derived formulations, such as multi-herbal extracts like Panchvalkal, suffer from batch variability due to inconsistent phytochemical profiles, leading to unpredictable ecological or toxicological outcomes [100].

Economic and manufacturing barriers further compound these concerns. Rare-earth nanoparticles (e.g., yttrium oxide nanosheets) and metallopharmaceuticals require resource-intensive synthesis protocols, raising costs and limiting accessibility in low-resource settings [156]. Hybrid delivery systems, such as bacteriophage-ZnO-NP conjugates, demand precise dosing regimens to balance efficacy and safety—a challenge that complicates clinical scalability [167].

### 6.7. Mechanistic Ambiguity

A fundamental barrier to developing QSI is the incomplete understanding of their modes of action, which stifles optimization and clinical translation. This mechanistic ambiguity arises when inhibitors interact with multiple targets or their primary biochemical interactions remain undefined, leading to unpredictable efficacy and off-target effects [338].

Natural product extracts exemplify this challenge. Multi-component formulations, such as those derived from *P. maculosa* or *B. officinalis*, exhibit synergistic anti-QS effects, but the contributions of individual phytochemicals are obscured. For instance, while these extracts reduce biofilm formation, the specific compounds responsible for LasR or RhlR inhibition are unidentified, complicating rational drug design [95]. Similarly, marine-derived *B. paralicheniformis* extracts require high concentrations for efficacy, yet their active principles remain uncharacterized, leaving researchers unable to isolate or enhance key molecules [257].

Synthetic and semi-synthetic compounds also suffer from unclear mechanisms. Tryptophan-containing cyclic dipeptides disrupt *P. aeruginosa* QS, but their precise molecular targets—receptor binding, signal molecule sequestration, or interference with transcriptional regulators—are poorly defined. This ambiguity hinders structure-activity relationship (SAR) studies, critical for potency optimization [336]. Bromotyrosine-derived inhibitors like psammaplin A further illustrate this issue. While they suppress *lasB* and *rhlA* gene expression, their variable efficacy across QS-regulated pathways (e.g., higher IC50 for *lasB* than *rhlA*) suggests non-specific interactions or off-target binding to bacterial metabolic enzymes [282].

Mycotoxin-derived compounds like citrinin introduce additional complexity by conflating anti-QS activity with host cell toxicity. Citrinin disrupts bacterial virulence at subinhibitory concentrations and impairs mitochondrial function in eukaryotic cells, implying dual mechanisms that are difficult to disentangle. This dual activity complicates dose optimization, as therapeutic windows narrow when antimicrobial and cytotoxic effects overlap [272].

Oxidative stress-inducing agents, including alkyl hydroperoxide E (AHE), exacerbate mechanistic uncertainty. While AHE inhibits QS by generating ROS, the exact pathways linking ROS to QS suppression—whether through signal molecule degradation, receptor oxidation, or transcriptional interference—are unresolved. This ambiguity risks unintended consequences, such as selecting antioxidant-rich bacterial mutants resistant to ROS and QS inhibition [339].

Fungal and actinobacterial extracts face reproducibility challenges due to uncharacterized bioactive components. For example, *T. polium* extracts fail to inhibit AHL-mediated QS in some studies, yet their variable phytochemical profiles across batches prevent definitive conclusions about their mechanism [94]. Similarly, *P. avium* cultivars exhibit inconsistent QS inhibition due to fluctuating polyphenol content, underscoring the need for standardized extraction protocols [85].

### 6.8. Partial Efficacy and Strain Dependency

The variable and incomplete suppression of virulence across *P. aeruginosa* strains or infection contexts represents a critical limitation of many QSI, undermining their reliability in clinical settings. Partial efficacy often stems from the redundancy of QS networks, where inhibitors targeting a single pathway fail to block alternative virulence mechanisms [340]. For instance, chrysin, a flavonoid, reduces pyocyanin production by only 41.4%, leaving significant residual virulence that sustains pathogenicity in chronic infections [341]. Similarly, isoeugenol achieves 70% biofilm inhibition at 400 μM—a concentration approaching cytotoxic thresholds—highlighting the narrow therapeutic window of many inhibitors [120]. Enzymatic inhibitors like AiiM lactonase exacerbate this issue by degrading AHLs without addressing downstream virulence effectors such as the T3SS, allowing *P. aeruginosa* to maintain cytotoxicity through non-QS pathways [171].

Strain dependency further complicates therapeutic outcomes. Farnesol, a natural sesquiterpene, exhibits reduced activity against mucoid *P. aeruginosa* phenotypes prevalent in CF airways. These strains overproduce alginate, a biofilm matrix component that physically shields QS receptors from inhibitor binding, rendering farnesol ineffective [129]. Similarly, plant-derived extracts from *D. americana* and *H. abdariffa* show discordant activity. While they inhibit pyocyanin production in vitro, their failure to reduce mortality in *C. elegans* infection models underscores the disconnect between biochemical assays and in vivo efficacy [90]. Such strain- or model-specific results challenge the extrapolation of preclinical data to human infections, where genetic and phenotypic diversity among clinical isolates is vast.

Biofilm heterogeneity amplifies these limitations. *P. aeruginosa* biofilms vary in matrix composition (e.g., alginate, Psl, Pel polysaccharides), and inhibitors targeting specific components, such as rhamnolipids or extracellular DNA, often fail against biofilms dominated by alternative polymers [342]. For example, recombinant lectin rHPLOE, which binds rhamnose residues, is ineffective against Pel-rich biofilms common in chronic wound infections [186]. This structural variability necessitates broad-spectrum inhibitors, yet most agents lack the versatility to adapt to dynamic biofilm architectures.

Compounding these issues, some inhibitors inadvertently exacerbate virulence. Malonate, while suppressing *lasR* expression, upregulates *pqsC* and increases pyocyanin production—a paradoxical effect that may worsen infection outcomes [138]. Similarly, pyranoanthocyanins enhance *pqsE* expression, potentially amplifying the production of cytotoxic factors [139].

### 6.9. Lack of In Vivo Validation

A critical translational gap for QSI is the overreliance on simplified in vitro or invertebrate models, leaving their efficacy and safety in mammalian systems inadequately characterized [343]. Many promising inhibitors fail to progress beyond early-stage preclinical testing due to insufficient validation in physiologically relevant in vivo environments. For instance, phage-derived proteins like PIT2 and synthetic peptides like Nesfactin have demonstrated robust anti-biofilm activity in vitro and invertebrate models (e.g., *C. elegans*) [181]. However, their performance in mammalian infection models—such as murine pneumonia or burn wound systems—remains unstudied, raising uncertainties about their pharmacokinetics, immunogenicity, or tissue penetration in complex host environments [178].

Natural product-derived inhibitors frequently suffer from this limitation. Psoralen, a coumarin derivative, reduces *P. aeruginosa* virulence in *C. elegans* but lacks validation in mammalian systems, where host-pathogen interactions, immune responses, and metabolic clearance pathways could diminish its efficacy [132]. Similarly, Boeravinone O, a flavonoid from *Boerhaavia diffusa*, exhibits potent QS inhibition in vitro but has not been tested in mammalian infection models, leaving its therapeutic potential speculative [101]. Even synthetic compounds like N-Aryl Malonamides (NAMs), despite showing efficacy in murine wound models, lack human clinical trials, leaving critical questions about dosage, toxicity, and bioavailability unanswered [344].

Nanoparticle-based strategies face similar translational hurdles. AgNPs and ZnC-NCs are often evaluated in in vitro biofilm assays or short-term invertebrate studies, bypassing chronic infection models that mimic human disease progression [345]. For example, proteomic analyses of AgNPs in *P. aeruginosa*-infected *Galleria* larvae reveal disrupted biofilm matrices. Still, these findings do not account for mammalian immune cell interactions or nanoparticle accumulation in organs like the liver or spleen [154]. Rare-earth nanoparticles (e.g., Y_2_O_3_ nanosheets) and bacteriophage-ZnO-NP conjugates, while effective in controlled lab settings, have not been assessed in large-animal models or human-relevant pharmacokinetic studies, stalling their clinical translation [156,167].

The consequences of this validation gap are profound. Invertebrate models, though cost-effective, lack the complexity of mammalian immune systems, tissue microenvironments, and metabolic pathways that influence drug efficacy. For instance, *C. elegans* cannot replicate the hypoxic conditions of CF lungs or the inflammatory milieu of chronic wounds, where *P. aeruginosa* biofilms thrive. Consequently, inhibitors like guanosine from *M. comosus*, validated only in *C. elegans*, may fail in human trials due to unanticipated host-pathogen interactions or rapid clearance by renal or hepatic systems [283,346].

### 6.10. Translational and Economic Barriers

The path to clinical adoption of QSI is fraught with economic, regulatory, and manufacturing challenges that extend beyond scientific limitations. Many promising compounds, particularly those derived from natural products or requiring complex synthesis, face prohibitive production costs [347]. For example, rare-earth nanoparticles like yttrium oxide nanosheets (Y_2_O_3_/NSs) demand resource-intensive synthesis protocols, including high-temperature calcination and specialized stabilizing agents, limiting scalability and affordability in low-resource healthcare settings [348]. Similarly, hybrid formulations such as SLNs co-loaded with antibiotics and QSI require precise control over lipid matrix stability during scale-up, often leading to batch variability and increased manufacturing complexity [15].

Regulatory hurdles further impede progress. Novel nanoparticle formulations or repurposed drugs must undergo rigorous safety and efficacy evaluations, which are complicated by the lack of standardized testing frameworks for antivirulence agents [349]. For instance, bacteriophage-ZnO-NP conjugates, while effective in lab settings, face uncertainty in regulatory approval due to their dual biological and inorganic components, necessitating extensive toxicological and environmental impact assessments [350]. Even well-characterized inhibitors like N-Aryl Malonamides (NAMs) struggle to advance to clinical trials without robust human pharmacokinetic data, as regulatory bodies prioritize compounds with established safety profiles [344].

Repurposed drugs, though economically attractive, introduce additional challenges. Ketoprofen derivatives, developed initially as anti-inflammatory agents, conflate QS inhibition with immune modulation, complicating regulatory approval due to ambiguous mechanisms of action [228]. Similarly, gamma irradiation—a cost-effective method to generate ROS for QS disruption—faces resistance from clinicians due to its non-specific tissue damage and lack of targeted delivery systems [268].

The economic viability of QSI is further strained by competition with conventional antibiotics, which dominate clinical practice despite rising resistance. Investors and pharmaceutical companies often prioritize antibiotics with immediate bactericidal effects over antivirulence agents, which are perceived as niche or adjunct therapies [351]. This bias stifles funding for large-scale trials, as seen with cyclic dipeptides or phage-derived proteins, which remain confined to academic research despite preclinical promise [178,336].

## 7. Future Perspectives

### 7.1. Structural Optimization and Multi-Target Inhibitor Design

The rational design of anti-QS molecules represents a cornerstone in combating *P. aeruginosa* resistance. It requires meticulous structural optimization to balance potency, specificity, and biocompatibility. This process integrates chemical synthesis, computational modeling, and synthetic biology to overcome the inherent limitations of early-generation QSI, such as poor pharmacokinetics, susceptibility to efflux pumps, and off-target cytotoxicity [352].

#### 7.1.1. Chemical Modifications for Enhanced Bioavailability and Target Affinity

Structural refinement of core pharmacophores is critical to improving drug-like properties. For instance, flavones and quinazolinones—natural scaffolds with intrinsic QS inhibitory activity—are chemically tailored to enhance solubility and membrane permeability [214]. Selenoester derivatives, where sulfur atoms in thiolactone rings are replaced with selenium, demonstrate superior stability against bacterial lactonases while retaining strong LasR antagonism [353]. Similarly, halogenation of brominated furanones (GBr) increases their electrophilic reactivity, enabling covalent binding to LasR’s ligand-binding domain and irreversible disruption of QS signaling [206]. Structure-activity relationship studies guide these modifications to minimize unintended interactions with host cells.

#### 7.1.2. Computational-Driven Design of Hybrid Inhibitors

Advances in in silico tools, such as molecular docking and molecular dynamics simulations, enable the systematic design of multi-target inhibitors [354]. For example, benzaldehyde derivatives are computationally optimized to bind conserved regions of both LasR and PqsR receptors, exploiting shared hydrophobic pockets to block autoinducer binding [124]. Machine learning algorithms further predict resistance-prone residues in QS receptors, guiding the synthesis of polymer-conjugated PqsR blockers with elongated alkyl side chains. These chains enhance biofilm penetration while avoiding recognition by efflux systems like MexAB-OprM [355]. Hybrid molecules, such as cephalosporin-QS inhibitor conjugates, exemplify dual functionality: the β-lactam core disrupts cell wall synthesis, while the appended furanone moiety inhibits RhlR-mediated virulence gene expression [194].

#### 7.1.3. Synthetic Biology and Efflux Pump Evasion Strategies

Synthetic biology approaches re-engineer existing antimicrobial scaffolds to evade resistance mechanisms. Modified N-acylcysteine derivatives are redesigned with bulky substituents (e.g., tert-butyl groups) that sterically hinder binding to efflux pump channels, prolonging intracellular retention [356]. Similarly, selenium-doped nanoparticles loaded with QSI exploit bacterial redox systems: selenite reduction generates ROSs that weaken biofilm integrity, while concurrently releasing inhibitors like halogenated furanones to silence LasR [159]. These strategies are complemented by substrate mimicry—designing inhibitors structurally analogous to natural QS autoinducers (e.g., 3-oxo-C12-HSL) to competitively saturate receptor sites without activating virulence pathways competitively [207].

### 7.2. Synergistic Therapies and Combinatorial Approaches

Synergistic combinations exploit multiple pathways to disrupt bacterial virulence and potentiate antimicrobial activity. For instance, QSI, such as thymoquinone, degrade biofilm EPS, exposing embedded bacteria to antibiotics. Thymoquinone reduces exopolysaccharide production by 70%, enabling colistin to penetrate dual-species biofilms (*S. aureus-P. aeruginosa*) and reduce bacterial load by 4-log units [131]. Concurrently, cationic polymers like chitosan bind to MexAB-OprM efflux pumps, blocking antibiotic extrusion and increasing intracellular ceftazidime concentrations by 15-fold [265]. Subinhibitory doses of azithromycin combined with the QSI furanone C-30 enhance macrophage phagocytosis by increasing P. aeruginosa’s sensitivity to host defense mechanisms, particularly nitrosative stress. This effect is partly attributed to the interference with *P. aeruginosa*’s QS systems, which regulate immune evasion mechanisms such as the production of virulence factors like *phzM* and p*v*dA [357]. Engineered phage-probiomic systems, such as bacteriophage-ZnO nanocomposites, degrade mature biofilms [156] while silencing *lasI* via CRISPR-dCas9 interference, achieving 3-log reductions in burn infection models [358].

### 7.3. Engineered Biological Systems and Nanotechnology

#### 7.3.1. Nanotechnology-Driven Delivery Platforms

NPs have emerged as versatile carriers for QSI, merging antivirulence and bactericidal functionalities. AgNPs functionalized with quorum-quenching enzymes, such as acylases, enzymatically degrade AHLs in biofilms while delivering bactericidal silver ions [152]. Hybrid nanomaterials like eugenol-conjugated AgNPs exploit hydrophobic interactions to dock into QS receptor pockets, blocking autoinducer binding and suppressing *lasI/rhlR* expression [305]. Similarly, ZnO-curcumin nanocomposites generate ROSs that disrupt LasR-mediated signaling while destabilizing biofilm matrices [158]. Innovations such as halogenated cerium oxide NPs mimic natural lactonase activity to address biocompatibility concerns, degrading QS signals without inducing oxidative damage to host tissues [162]. Stimuli-responsive nanocarriers further refine delivery; pH-sensitive liposomes release encapsulated caraway essential oils in acidic biofilm microenvironments, ensuring localized and sustained anti-QS activity [359].

#### 7.3.2. Inhalable and Topical Formulations

Pulmonary and chronic wound infections demand site-specific delivery systems. Inhalable dry powder formulations, such as lactonase-loaded chitosan microparticles, target *P. aeruginosa* biofilms in CF airways, reducing *lasB* elastase production by 80% in preclinical models. These systems leverage mucoadhesive polymers to prolong residence time in the bronchial mucosa [360,361]. For chronic wounds, collagen-based hydrogels embedded with cytotoxic peptides (e.g., G3KL) enable localized delivery, disrupting biofilms without harming regenerating epithelial cells [362,363]. Cyclodextrin complexes sequester hydrophobic QSI, like hamamelitannin, enhancing solubility and enabling controlled release in exudative wound environments [364].

### 7.4. Translational Challenges and Ecological Integration

#### 7.4.1. Preclinical Validation: Bridging the Gap Between Bench and Bedside

Robust preclinical models must replicate the polymicrobial, chronic infection landscapes seen in clinical settings. For instance, rodent catheter-associated biofilm models incorporate co-infections with *Candida albicans* and *S. aureus* to mimic diabetic foot ulcer microbiomes, enabling the evaluation of compounds like allopurinol-metformin hybrids under realistic conditions. These models assess biofilm eradication and host-pathogen dynamics, such as neutrophil recruitment and cytokine profiling [365,366]. Pharmacokinetic/pharmacodynamic (PK/PD) modeling is critical for optimizing dosing regimens; thymoquinone nanoformulations, for example, require adjusted dosing intervals in CF models due to altered mucus clearance rates [131]. Safety assessments extend beyond acute toxicity, with 90-day murine studies revealing that selenium-doped nanoparticles induce hepatic glutathione depletion at high doses, necessitating formulation refinements [367].

#### 7.4.2. Standardization and Reproducibility

Natural product-derived QSI, such as propolis triterpenes and *B. diffusa* extracts, suffer from batch-to-batch variability due to seasonal and geographical factors [101,145]. Advanced phytochemical fingerprinting via HPLC-MS and AI-driven cultivation protocols is being deployed to standardize bioactive compound ratios [368]. Synthetic biology offers an alternative; engineered *Saccharomyces cerevisiae* strains now produce uniform yields of hamamelitannin analogs, achieving >95% purity for clinical-grade batches [369].

#### 7.4.3. Ecological and Evolutionary Considerations

The ecological ripple effects of anti-QS therapies remain understudied. Horizontal gene transfer (HGT) risks are amplified in wastewater ecosystems, where engineered phages or plasmid-encoded QSI could disseminate resistance genes [370]. Metagenomic surveillance of hospital effluents post-trial revealed *lasR* mutations in non-target *Acinetobacter* spp., highlighting the need for biodegradable inhibitors [10]. For example, PLGA nanoparticles loaded with brominated furanones hydrolyze into lactic and glycolic acids, reducing environmental persistence [371,372]. Evolutionary trade-offs must also be managed: longitudinal exposure assays show that *P. aeruginosa* develops compensatory mutations in *rhlR* when treated with LasR-specific inhibitors, necessitating multi-target approaches [373]. CRISPR-Cas9-based allele-specific silencing of mutant *lasR* variants is being explored to preempt resistance [374].

#### 7.4.4. Regulatory and Commercialization Hurdles

Regulatory agencies lack standardized guidelines for anti-QS therapies, particularly combination products like phage-nanoparticle conjugates. The FDA’s Complex Drug Advisory Committee now requires ecotoxicology dossiers for nanotherapeutics, including biofilm dispersal impacts on aquatic ecosystems [375]. Commercial scalability is another bottleneck; the Good Manufacturing Practice (GMP) production of stimuli-responsive nanocarriers costs 3–5 times more than conventional antibiotics, limiting low-resource market access [376]. Public-private partnerships, such as the CARB-X-funded “QuorumSafe” initiative, subsidize early-stage development to offset these barriers [377].

#### 7.4.5. Future Directions: Toward Sustainable Anti-Infective Strategies

Next-generation translational frameworks prioritize One Health integration. Phase IV post-marketing surveillance will monitor long-term ecological impacts, using blockchain-enabled supply chains to track environmental dispersal of QSI [132]. Personalized medicine approaches, guided by patient-specific *lasR* SNP profiles and microbiome signatures, aim to optimize inhibitor-antibiotic pairings [378]. Meanwhile, “green” formulation strategies—such as algae-based chitosan production and solar-driven nanoparticle synthesis—reduce anti-QS therapeutics’ carbon footprint [379].

### 7.5. Mechanistic Insights and Resistance Management

Multi-target inhibition strategies are paramount to preempting resistance. Hybrid molecules such as efflux pump inhibitors, PAβN derivatives, covalently modify both LasR and MexAB-OprM efflux pumps, reducing virulence while enhancing intracellular antibiotic retention [192]. Natural polysaccharides like ulvan from green algae employ a dual mechanism—sequestering AHL signals via lactonase-mimetic activity and downregulating *pqsABCDE* through zinc ion chelation [380]. Synthetic biology approaches further enable “resistance-proof” therapies; engineered phages delivering dCas9-sgRNA complexes silence *lasR* and *mexT* simultaneously, suppressing compensatory pathway activation [381]. Combinatorial regimens, such as pairing PqsR inhibitors with tobramycin-loaded nanoparticles, exploit collateral sensitivity, where *pqsR* mutants exhibit 100-fold increased susceptibility to aminoglycosides due to disrupted membrane potential regulation [382].

## 8. Conclusions

Developing anti-QS therapies against *P. aeruginosa* is a promising yet complex endeavor constrained by biological redundancy, pharmacokinetic inefficiencies, and evolutionary adaptability. Overcoming these challenges requires a multidisciplinary approach, integrating structural chemistry, systems biology, and translational medicine. Multi-target inhibitors can bypass redundancy, advanced delivery systems enhance bioavailability, and rigorous in vivo validation ensures safety and efficacy. By harmonizing molecular optimization, synergistic combinations, and innovative delivery, these strategies promise to redefine infection management. Collaborative efforts across microbiology, pharmacology, and nanotechnology will translate anti-QS molecules from experimental tools into clinically viable solutions, offering a sustainable paradigm in the post-antibiotic era and curbing *P. aeruginosa* resistance.

## Figures and Tables

**Table 1:** Plant-based inhibitors of QS in *P. aeruginosa*.

Inhibitor Name	Plant/Source	Target QS Component
**Diallyl Disulfide (Compound 2i)**	*Allium sativum*	LasR
**Astragaloside IV**	*Astragalus membranaceus*	MexAB-OprM efflux pump
**Ortho-Vanillin**	*Benzaldehyde derivative*	RhlR
**Berberine**	Berberis species	PqsA
***Bistorta officinalis* (BIO)**	*Bistorta officinalis*	LasR
**Boeravinone O**	*Boerhavia diffusa*	LasR
**Cajaninstilbene Acid (CSA) 3o**	*Cajanus cajan*	*lasB, pqsA*
**Carvone (Caraway EO)**	*Carum carvi* (*Caraway*)	LasR, CviR
**Lutein**	*Chlorella pyrenoidosa* (*Algae*)	LasI, LasR, RhlI, RhlR
**Caffeine**	*Coffea arabica* (*Coffee*)	LasR, LasI
**Sulforaphane**	*Cruciferous vegetables* (e.g., *broccoli*)	Lux-type receptors (TraR, QscR, CviR)
**Bakuchiol**	*Cullen corylifolium*	PqsR
***Diphysa americana* Extract**	*Diphysa americana*	QS-regulated virulence factors
**Panchvalkal (Polyherbal)**	*Ficus* spp. and *Albizzia lebbec*	Nitric oxide reductase (NOR)
**Phillyrin**	*Forsythia suspensa*	QS signaling network
***Fragaria vesca* Extract**	*Fragaria vesca* (*Wild strawberry*)	AI-1, AI-2 signaling
**6-Gingerol**	*Ginger* (*Zingiber officinale*)	QscR, LasR, PqsR, RhlR
***Hibiscus sabdariffa* Extract**	*Hibiscus sabdariffa*	QS-regulated virulence factors
**Compound 5f (Ligustilide derivative)**	*Ligusticum chuanxiong* (*Chinese lovage*)	LasR/LasB
**Mangiferin Derivatives**	*Mangifera indica* (*Mango*)	LasR
**Methyl Gallate (MG)**	*Mangifera indica* (*Mango*)	LasR, LasI
**1,8-Cineole**	*Musa paradisiaca* (*Banana*)	Las, Rhl systems
**Thymoquinone**	*Nigella sativa* (*Black seed*)	LasR, RhlR, PqsR
**Falcarindiol**	*Notopterygium incisum*	lasI, lasR, rhlI, rhlR, lasB
**Carvacrol**	*Oregano*, *Thyme*	LasI, LasR, BswR
**Paeonol**	*Paeonia suffruticosa* (*Tree peony*)	Las, Rhl, Pqs systems
**American Ginseng Extract (AGE)**	*Panax quinquefolius*	Swarming, swimming motility
***Persicaria maculosa* (PEM)**	*Persicaria maculosa*	LasR
**Psoralen**	*Psoralea corylifolia*	LasR, RhlR, PqsR
***Salvia sclarea* Essential Oil**	*Salvia sclarea*	Las, Rhl systems
**Cinnamoyl Hydroxamates (CHA, MCHA)**	Synthetic (*Cinnamoyl derivatives*)	RhlI/R system
**Coumarin-Chalcone C9**	Synthetic (Coumarin-Chalcone conjugate)	LasR, PqsR
**Isovanillin**	Synthetic/Vanilla derivatives	*las, pqs* systems
***Syzygium aromaticum* Extract**	*Syzygium aromaticum* (*Clove*)	QS signal molecules
**Trigonelline Hydrochloride**	*Trigonella foenum-graecum* (*Fenugreek*)	Las, Rhl, Pqs pathways
**Wild Blueberry Extract**	*Vaccinium angustifolium*	*lasI, lasR, rhlI, rhlR*
**Vanillin**	*Vanilla planifolia*	PqsR
**Quercetin**	Various plants (e.g., onions, apples)	QS proteins
**Ginger Extract**	*Zingiber officinale*	*lasI, lasR, rhlI, rhlR*

**Table 2:** Nanoparticle-based inhibitors of QS in *P. aeruginosa*.

Inhibitor Name	Molecule Used in Nanoparticle	Target Component of QS
**P-AgNPs**	Silver nanoparticles (propolis extract)	AHL-mediated signaling pathways
**AgCS@AC NPs**	Silver nanoparticles, chitosan, acylase I	AHLs (degradation)
**AgNPs (*Eruca sativa*)**	Silver nanoparticles	Pyocyanin, LasA/B proteases, exopolysaccharides (QS-regulated virulence factors)
**AgNPs (*Ducrosia flabellifolia*)**	Silver nanoparticles	AHLs, Las/Rhl systems
**AgNPs**	Silver nanoparticles	QS-related genes, PQS biosynthesis, virulence factors (pyocyanin, elastase, rhamnolipids)
**SBT@AgNPs**	Silver nanoparticles (Seabuckthorn extract)	AHLs (degradation)
**AgNP + 4NPO**	Silver nanoparticles, 4-nitropyridine N-oxide	Las, Rhl, Pqs systems
**Pb-AgNPs**	Silver nanoparticles (Piper betle extract, eugenol)	LasR, LasI, MvfR
**ZnO-NPs (orange peel)**	Zinc oxide nanoparticles	*lasI* gene (AHL synthase)
**Zinc Oxide Nanospikes (ZNs)**	Zinc oxide	Las, Rhl, Pqs systems
**ZnC-NCs**	Zinc oxide/curcumin nanocomposites	LasR, RhlR
**Eugenol_Au NPs**	Gold nanoparticles (eugenol)	QS signaling pathways, ROS-mediated disruption
**Fe_3_O_4_@EUG**	Magnetite nanoparticles (eugenol)	QS signaling (exact target unspecified)
**SLNs (Ofloxacin + Eugenol)**	Solid lipid nanoparticles (eugenol)	QS-regulated virulence factors
**Eugenol nanoemulsion**	Eugenol nanoemulsion	*lasI*, *rhlI* genes
**SeNPs**	Selenium nanoparticles	LasI, LasR, RhlI, RhlR, MvfR
**SeNPs**	Selenium nanoparticles	AHL biosynthesis
**TDN**	Titanium dioxide nanoparticles	*lasI*, *lasR*, *rhlI*, *rhlR*, *pqsA*, *pqsR* genes
**mBTL-CANPs**	Calcium alginate nanoparticles (meta-bromo-thiolactone)	LasR, RhlR
**CeO_2_ NPs**	Cerium dioxide nanoparticles	AHL-mediated QS (via halogenated compounds)
**QSINPs (ajoene)**	Chitosan/dextran sulfate nanoparticles (ajoene)	QS-regulated virulence factors (pyocyanin, rhamnolipids)
**Y_2_O_3_ nanospheres**	Yttrium oxide nanoparticles	AHL-mediated QS
**Zeolite 4A**	Zeolite 4A	Adsorption of AHLs (3-oxo-C6-HSL, 3-oxo-C12-HSL)

**Table 3:** Examples of peptides, enzymes, and proteins inhibiting QS in *P. aeruginosa*.

Inhibitor Name	Type	Origin	QS Factor Inhibited
**Micafungin**	Lipopeptide	Semi-synthetic	Biofilm-related genes (alginate, 1,3-β-D-glucan, pellicles); QS virulence factors
**Nesfactin**	Lipopeptide	*Nesterenkonia* sp. MSA31	*las* and *rhl* systems (via AHL degradation: 3-oxo-C12-HSL, C4-HSL)
**CRAMP**	Peptide	Host (cathelicidin-related)	*lasR, lasI, rhlR, rhlI*
**WSF, FASK, YDVD**	Peptide	Synthetic	LasR
**Cyclo(Pro-Tyr)-like cyclic dipeptides**	Peptide	Synthetic	LasR, Rhl systems
**G3, C8G2**	Peptide	Synthetic	LasR
**β-peptide polymer (20:80-Bu:DM)**	Peptide	Synthetic	*las, rhl, pqs* systems
**PA-Win2**	Peptide	Pardosa astrigera (spider)	Las, Pqs, Rhl systems
**Tryptophan-containing CDPs**	Peptide	Synthetic	*rhl, pqs* systems
**LIVRHK, LIVRRK**	Peptide	Synthetic	*lasI, lasR, rhlI, rhlR*
**Peptide mix (Olivancillaria hiatula)**	Peptide	Olivancillaria hiatula (marine snail)	QS-regulated virulence factors (biofilm, pyoverdine, pyocyanin, proteases)
**PIT2**	Protein	*Pseudomonas* phage LMA2	*lasR*, *lasI, rsaL, prpL, lasA, pqsH*
**YtnP**	Protein	*Bacillus velezensis*	AHLs (Las and Rhl systems)
**Aqs1**	Protein	*Pseudomonas* phage DMS3	LasR
**AiiM lactonase**	Protein	Bacterial (source unspecified)	AHLs (Las, Rhl systems)
**rHPLOE**	Protein	Tachypleus tridentatus (horseshoe crab)	Rhl system components (di-rhamnolipids, Psl)
**AidB**	Protein	*Bosea* sp.	AHLs (Las, Rhl systems)
**MomL**	Protein	Marine-derived (expressed in *B. brevis*)	AHLs (Las, Rhl systems)
**AiiA lactonase KMMI17**	Protein	Bacillus thuringiensis KMCL07	AHLs (Las, Rhl systems)

**Table 4:** Examples of synthetic and derivative molecules inhibiting QS in *P. aeruginosa*.

Inhibitor Name	Type of Inhibitor	Chemical/Pharmaceutical Class	QS Factor(s) Inhibited
Phosphate ester derivative of chrysin	Allosteric LasR inhibitor	Flavonoid derivative	LasR
Halogenated phenazine compounds		Phenazine derivatives	LasR
1,3,4-oxadiazoles (15, 23)	Competitive antagonist	1,3,4-oxadiazoles	PQS system
6f (1H-benzimidazole)		1H-benzimidazole-based	PqsR
QZN 34		3-C3NH2-7Cl-C9-QZN derivative	PqsR
Propyl gallate (PG)		Alkyl gallate	LasR, RhlR
Compound **30**		Alkynyl ketone	RhlR
Y-31		Amino-tetrahydro-oxazinone	LasR, RhlR, PqsR
Paracetamol		Analgesic (NSAID)	LasR
Cilostazol		Antiplatelet/vasodilator	LasR, RhlR
4-hydroxybenzaldehyde, vanillin, syringaldehyde		Benzaldehydes	LasR
Benzimidazolium salts (2, 3)		Benzimidazolium salts	LasR
6b		Benzoheterocyclic sulfoxide derivative	LasR
Benzothiazole-2-thiol (7)		Benzothiazole derivative	LasB
GBr		Brominated furanone	LasR
Cefoperazone		Cephalosporin antibiotic	PQS system
Ceftriaxone (CRO) and CRON		Cephalosporin antibiotics	LasR, LasI, PqsR
6n (chromone-2-carboxamide)		Chromone-2-carboxamide	PqsR
L-HSL		Cyclic butyrolactone derivative	LasR, RhlR, PqsR
Sitagliptin		DPP-4 inhibitor	LasR, RhlR
Furanone C-30		Furanone	LasR, RhlR
23^e^ (4-fluorophenyl-furanone)		Furanone derivative	Las, Rhl
5-HMF		Hydroxymethylfurfural	LasR, RhlR
Itaconimides (e.g., 18a)		Itaconimide	LasR, RhlR, PqsR
NOI, NDI		N-alkylimidazole derivatives	LasR
D88 (N-Aryl Malonamide)		N-Aryl Malonamide (NAM)	PqsR
Secnidazole		Nitroimidazole antibiotic	lasI, lasR, rhlI, rhlR, pqsA, pqsR
Dimetridazole, Ribavirin		Nitroimidazole, nucleoside analog	LasR, RhlR, PqsR
Ibuprofen		NSAID	Rhl system (C4-HSL)
Ketoprofen, G20		NSAID derivatives	PqsR
5b (phenyloxadiazole sulfoxide)		Phenyloxadiazole sulfoxide	LasR
P2-QSI (polymer conjugate)		Polymer-PqsR inhibitor conjugate	PqsR
5d (N-pyridinylbenzamide)		Pyridinylbenzamide derivative	Las/Rhl
Ketone-/cyano-selenoesters		Selenoesters	AI-1/AI-2 pathways
PqsR inhibitors (small molecules)		Small molecules	PqsR
Sulfamethoxazole-silver complex (SMTZAg)		Sulfonamide-metal complex	LasR
7g		Thioether-linked dihydropyrrol-2-one	PqsR
Allopurinol		Xanthine oxidase inhibitor	LasR, RhlR
Propranolol		β-adrenergic receptor blocker	*lasR, rhlR, pqsR*
Cephalosporins (cefepime, ceftazidime, ceftriaxone)		β-lactam antibiotics	LasR, PqsR, CviR
Triazole nucleoside mimics		Triazole nucleoside	PqsA
Thiazolo-indolin-2-one derivatives (7a, 4, 12)	Dual PqsR/DHFR inhibitor	Thiazole-indole hybrids	PqsR
10d		3-hydroxy-1,6-dimethylpyridin-4-one	FpvA receptor
Levamisole	QS gene downregulator	Anthelmintic	*lasI, lasR, rhlI, rhlR, lasB*
Chloroquine		Antimalarial	*lasI, lasR, rhlI, rhlR, lasB*
Sodium New Houttuyfonate (SNH)		Fatty acid derivative	*rhlI, pqsA*
Erythromycin		Macrolide antibiotic	*lasI, lasR, rhlI, rhlR, pqsA, pqsR*
Furazolidone		Nitrofuran antibiotic	*lasR, rhlR, pqsR*
Ostarine		Selective androgen receptor modulator (SARM)	*lasR, rhlI, rhlR, pqsA-E, pqsH*
trans-Cinnamaldehyde (CA)		Aldehyde	*lasI, lasR, rhlI, rhlR*
Guanfacine		Antihypertensive	QseC/QseB homolog
1,3-DMP		Diamine	lasR
Compound **11f** (L-homoserine analog)		L-homoserine lactone analog	*lasI, lasR, rhlI, rhlR, pqsA, pqsR*
Furazolidone		Nitrofuran antibiotic	*lasR, rhlR, pqsR*
HMMN		Nitroimidazole	*lasR, rhlR, pqsR*
NAC derivatives (4d, 4h)	LasR antagonist	N-Acylcysteine derivatives	LasR
OBS 9a, 9b, 9f		Oxazolone-sulfonamide hybrids	LasR
2-Difluoromethylpyridine derivatives (1, 5, 6)		Pyridine derivatives	LasR
Chalcone derivatives (5H, Nme2)	PqsR antagonist	Chalcones	PqsR
Compound **61**		Quinazolin-4(3H)-one derivative	PqsR
Quinazolinone derivatives (11a, 11b)		Quinazolinone-based compounds	PqsR
Clofoctol		Chlorinated phenolic ether	PqsR
HIPS-1635		Nanoparticle-in-liposome formulation	PqsR
Thiazole-based QSIs (27, 34)		Thiazole derivatives	PqsR
Lamivudine (LAM)	QS receptor binder	Nucleoside analog	LasR, RhlR, PqsR
Metformin and Vildagliptin		Antidiabetic drugs	LasR, RhlR, PqsR
Doxazosin	QS protein antagonist	α-adrenoreceptor blocker	QseC, PmrA
Terazosin	QscR antagonist	α-adrenoreceptor blocker	QscR
β-Blockers (atenolol, esmolol, metoprolol)	QscR stabilizer	β-adrenergic receptor blockers	QscR
Azithromycin (AZM)	AHL production inhibitor	Macrolide antibiotic	AHL synthesis
MHY1383, MHY1387	Non-competitive QSI	Synthetic small molecules	LasR, QscR, RhlR
Nitrofurazone	PqsE inhibitor	Nitrofuran antibiotic	PqsE

**Table 5:** Some examples of inhibitors produced by microorganisms targeting QS in *P. aeruginosa*.

Inhibitor Name	Producing Species	Nature of Inhibitor	QS Factor(s) Inhibited
**Metabolites (MELF)**	*Aspergillus quandricinctus*	Fungal metabolites	AHL-mediated QS systems
**Amylomacrolactines A–C**	*Bacillus amyloliquefaciens* SCSIO 41392	Macrolactins	PQS system, pyocyanin production
**Diketopiperazines (DKPs)**	*Bacillus cereus* RC1	Diketopiperazines	QS-regulated virulence factors (pyocyanin, motility)
**AHL-degrading enzymes**	*Bacillus cereus*, *Bacillus subtilis*, *Pseudomonas* spp.	Enzymes (lactonases, acylases)	AHL molecules
**AHL-degrading enzymes**	*Bacillus* spp., *Vibrio alginolyticus*	Enzymes (lactonases, acylases)	AHL molecules
**Phthalate derivative**	*Bacillus zhangzhouensis* SK4	Phthalate derivative	LasR
**Fungal extract**	*Blastobotrys parvus* PPR3	Fungal extract	LasR, RhlR
**Cladodionen**	*Cladosporium* sp. Z148	Fungal metabolite	LasR, PqsR
**C18-HSL analogs**	*Delftia tsuruhatensis* 11304	AHL analogs	Las, Rhl, Pqs systems
**Hydrocinnamic acid**	*Enterobacter xiangfangensis*	Organic acid	*lasI*, *lasR*, *rhlI*, *rhlR*
**3-Benzyl-hexahydro-pyrrolo[1,2-a]pyrazine-1,4-dione**	*Exiguobacterium indicum* SJ16	Cyclic dipeptide	*las*, *rhl*, *pqs* systems
**Glabrol, Berberine, Fucoxanthin**	Halophilic bacteria (e.g., *Halomonas* spp.)	Bioactive metabolites	*las*, *rhl* systems
**Biosurfactant**	*Lactiplantibacillus plantarum*	Biosurfactant	AHL signaling
**Cell-free extract**	*Lactobacillus plantarum* F-10	Cell extract	QS-regulated virulence factors (*las*, *rhl* systems)
**Lyngbyoic acid, Benderadiene**	*Lyngbya* sp.	Specialized metabolites	LasR, RhlR
**AHL-degrading enzymes**	Marine actinobacteria	Enzymes (lactonases, acylases)	AHL molecules
**Chitosan**	*Metapenaeus affinis*	Polysaccharide	*lasR*, *rhlR*
**Secondary metabolites**	*Nocardiopsis lucentensis* EMB25	Secondary metabolites	LasR, RhlR
**2-Methyl-N-(2′-phenylethyl) butyramide, etc.**	*Oceanobacillus* sp. XC22919	Butyramides, benzoate	AHL-mediated QS systems
**Chermesiterpenoid B (Che B) and Che B Ester**	*Penicillium chermesinum*	Terpenoids	LasR, QscR
**Tyrosol**	*Penicillium chrysogenum* DXY-1	Phenolic compound	CviR (AHL receptor)
**Citrinin**	*Penicillium* sp. JH1	Secondary metabolite	*lasI*, *lasR*, *rhlI*, *rhlR*, *pqsA*, *pqsR*
**Curvularin**	*Phoma macrostoma*	Aromatic compound	RhlR
**1-(4-amino-2-hydroxyphenyl)ethanone (AHE)**	*Phomopsis liquidambari*	Fungal metabolite	*lasI*, *lasR*, *rhlI*, *rhlR*, *pqsR*
**VOC-3.9**	*Spongiibacter nanhainus* CSC3.9	Volatile organic compounds	LasR, RhlR, PqsB, AmbB
**Actinomycin D**	*Streptomyces cyaneochromogenes* RC1	Antibiotic	*lasI*, *lasR*, *rhlI*, *rhIR*, *pqsR*
**Fatty acyl compounds (e.g., 13Z-Octadecenal)**	*Streptomyces griseoincarnatus* HK12	Fatty acyl compounds	LasI
**Tyramine, N-acetyltyramine**	*Vibrio alginolyticus* M3-10	Amines	AHL-mediated QS systems

**Table 6:** Comparative summary of QSI classes.

Inhibitor Class	Representative Compound(s)	QS Target	Reported Activity Metric	Experimental System	Key Limitations
**Microbial-Derived**	Amylomacrolactine A, Citrinin	PQS, LasR/RhlR	Pyocyanin inhibition: 68.64% (Citrinin)	*P. aeruginosa* PAO1, clinical strains	Narrow-spectrum, production complexity
	Curvularin	RhlR	Rhamnolipid reduction: 34.7%	*P. aeruginosa* PAO1, *C. elegans*	Weak LasR inhibition
**Plant-Derived**	Thymoquinone, 6-Gingerol	LasR, RhlR, PqsR	Biofilm inhibition: 42.74% (6-Gingerol)	*P. aeruginosa* PAO1, murine models	Poor solubility, metabolic instability
	Falcarindiol, Bakuchiol	LasR, PqsR	Elastase reduction: 35.7% (Falcarindiol)	Burned mouse model	Dose-dependent cytotoxicity
**Synthetic Compounds**	Furanone C-30, Compound **5b**	LasR, RhlR	IC_50_: 8.7 μM (Compound **5b**)	*P. aeruginosa* PAO1, *C. elegans*	Hemolysis at high doses
	Benzoheterocyclic sulfoxide (6b)	LasR	Biofilm inhibition: 40% at 300 μg/mL	*P. aeruginosa* PAO1	Limited in vivo data
**Peptides**	CRAMP, PA-Win2	QS-regulated genes	Biofilm reduction: 91.05% (CRAMP)	*P. aeruginosa* biofilm assays	Proteolytic degradation
	LIVRHK/LIVRRK peptides	LasI, RhlI	Virulence factor reduction: 61% (pyocyanin)	*P. aeruginosa* PAO1	Short half-life in vivo
**Nanoparticles**	AgNPs (propolis), ZnO-NPs	AHL degradation, LasR	Violacein inhibition: 75.24% (AgNPs)	*C. violaceum* CV026, *P. aeruginosa*	Cytotoxicity, environmental impact
	Eugenol-functionalized NPs	LasR, RhlR	Pyocyanin reduction: 66%	Carbapenem-resistant *P. aeruginosa*	ROS-mediated host cell damage
**Enzymes**	AiiA lactonase, MomL	AHL hydrolysis	Elastase reduction: >50% (AiiA)	*P. aeruginosa* clinical strains	Instability in physiological fluids
	YtnP lactonase	AHL degradation	Biofilm inhibition: 90%	Simulated water filter system	Thermolability

## Data Availability

The original contributions presented in the study are included in the article, further inquiries can be directed to the corresponding author.
